# Heterologous Expression and Biochemical Characterization of Two Lipoxygenases in Oriental Melon, *Cucumis melo* var. *makuwa* Makino

**DOI:** 10.1371/journal.pone.0153801

**Published:** 2016-04-21

**Authors:** Songxiao Cao, Hao Chen, Chong Zhang, Yufan Tang, Jieying Liu, Hongyan Qi

**Affiliations:** Key Laboratory of Protected Horticulture of Education Ministry and Liaoning Province, College of Horticulture, Shenyang Agricultural University, Shenyang, Liaoning, China; Southern Illinois University School of Medicine, UNITED STATES

## Abstract

Lipoxygenases (LOXs) are a class of non-heme iron-containing dioxygenases that catalyse oxidation of polyunsaturated fatty acids to produce hydroperoxidation that are in turn converted to oxylipins. Although multiple isoforms of LOXs have been detected in several plants, LOXs in oriental melon have not attracted much attention. Two full-length LOX cDNA clones, *CmLOX10* and *CmLOX13* which have been isolated from oriental melon (*Cucumis melo* var. *makuwa* Makino) cultivar “Yumeiren”, encode 902 and 906 amino acids, respectively. Bioinformatics analysis showed that CmLOX10 and CmLOX13 included all of the typical LOX domains and shared 58.11% identity at the amino acid level with each other. The phylogenetic analysis revealed that CmLOX10 and CmLOX13 were members of the type 2 13-LOX subgroup which are known to be involved in biotic and abiotic stress. Heterologous expression of the full-length CmLOX10 and truncated CmLOX13 in *Escherichia coli* revealed that the encoded exogenous proteins were identical to the predicted molecular weights and possessed the lipoxygenase activities. The purified CmLOX10 and CmLOX13 recombinant enzymes exhibited maximum activity at different temperature and pH and both had higher affinity for linoleic acid than linolenic acid. Chromatogram analysis of reaction products from the CmLOX10 and CmLOX13 enzyme reaction revealed that both enzymes produced 13S-hydroperoxides when linoleic acid was used as substrate. Furthermore, the subcellular localization analysis by transient expression of the two LOX fusion proteins in tobacco leaves showed that CmLOX10 and CmLOX13 proteins were located in plasma membrane and chloroplasts respectively. We propose that the two lipoxygenases may play different functions in oriental melon during plant growth and development.

## Introduction

Lipoxygenases (LOX; linoleate: oxygen oxidoreductase, EC 1.13.11.12) are a class of non-heme iron-containing dioxygenases that catalyze oxidation of polyunsaturated fatty acids (PUFAs) with a (Z, Z)-1,4-pentadiene structure to produce unsaturated fatty acid hydroperoxides [1]. They are widely distributed throughout the plant, animal, and microorganism kingdoms [2–5]. Plant LOXs are ubiquitous and encoded by multigene families [6]. The metabolism of PUFAs via the LOX-catalyzed step and the subsequent reactions are collectively named LOX pathway which contains at least seven multienzyme branches [1]. LOXs initiate the synthesis of diverse compounds collectively called oxylipins including jasmonates, leaf aldehydes and alcohols, which have diverse functions in plants [1,7,8].

Based on the positional specificity of the oxygen insertion, plant LOXs are grouped into 9-LOXs and 13-LOXs which produce 9- and 13-hydroperoxides respectively. Both hydroperoxides can be enzymatically cleaved to aldehydes and ω-oxo acids [9] and the 13-hydroperoxide of linolenic acid can be converted to jasmonic acid [10]. According to primary structure and overall sequence similarity, plant LOXs can be further divided into two subfamilies, type 1 and type 2 LOXs. Type 1 LOXs harbor no plastidic transit peptide and have a high sequence similarity (>75%) to each other. In contrast, type 2 LOXs carry a plastidic transit peptide and have a moderate sequence similarity about 35% to each other [1]. However, a few nontraditional plant LOXs can oxygenate at C-9 and C-13 positions and produce both 9- and 13- hydroperoxides, such as tea plant CsLOX1, olive LOX and rice OsLOX1 [11–13]. Through the heterologous expression in *Escherichia coli*, positional specificity of LOXs in diverse plants was identified. Pepper CaLOX1 encodes a 9-specific lipoxygenase [14], while tomato TomLoxF produces 13-hydroperoxide derivatives [15]. In addition, rice OsLOX1 and pea LOXN2 have dual positional specificity [13,16].

In plants, LOXs are related to many biological processes and physiological functions, such as tuber development [17], generation of fruit flavor volatiles [18], resistance against pathogens [13], sex determination [19] and senescence [20]. LOX isoforms have different preferences with substrate, positional specificity of substrate oxygenation and subcellular localization [1]. The subcellular localization of LOX proteins provides some help for predicting the physiological functions of different LOX isoforms in plants. Besides plastid and cytoplasm, particulate LOXs are also found in microsomal membranes, plasma membranes, the cytosol and vacuole and of leaf chloroplast envelopes [1]. LOX isoforms which have different locations may have different functions during plant growth and development. In plants, LOX gene expression is regulated by different stress such as wounding [21,22], pathogen infection [23] or different signaling molecules such as methyl jasmonate (MeJA) and salicylic acid (SA) [23–25] which are well-known modulators of defense responses. Expression analysis of *PvLOX6* in common bean showed that wounding or non-host pathogen infections, as well as signaling molecules like H_2_O_2_, SA and MeJA regulated *PvLOX6* expression [21].

The oriental melon (*Cucumis melo* var. *makuwa* Makino) is an important agricultural commodity and widely grown in China and eastern Asian countries. In comparison with other plants, there is relatively little known about oriental melon lipoxygenases. With the completeness of melon genomic sequencing project which provides an ideal way for melon gene cloning and functional analysis [26], we have identified 18 candidate LOX genes in the melon genome [27]. In this study we have isolated intact *CmLOX10* and *CmLOX13* cDNAs which they get together closely in phylogenetic tree from the tender stem of 30-day-old oriental melon (*Cucumis melo* var. *makuwa* Makino) cultivar “Yumeiren” and expressed CmLOX10 and CmLOX13 in *Escherichia coli* to characterize the two proteins. The results show that the recombinant CmLOX10 and CmLOX13 preferentially consume linoleic acid and introduce oxygen onto the 13th carbon of the fatty acid. The subcellular localization analysis by transient expression of CmLOX10 and CmLOX13 in tobacco leaves shows that CmLOX10 and CmLOX13 proteins are located in plasma membrane and chloroplasts respectively. This study provides knowledge on the physiological function of *CmLOX10* and *CmLOX13* genes in oriental melon.

## Materials and Methods

### Plant materials

Oriental melon (*Cucumis melo* var. *makuwa* Makino) cultivar “Yumeiren” were individually grown in a greenhouse at 25 ± 2°C, under a 14-h light/10-h dark photoperiod at Shenyang Agricultural University in China. The tender stems of 30-day-old oriental melon were immediately frozen in liquid nitrogen and stored at -80°C until used for the cloning of *CmLOX10* and *CmLOX13*. Tobacco (*Nicotiana benthamiana*) plants for agro-infiltration were raised in a growth chamber at 25°C under a 16/8 h day/night cycle for six weeks.

### Isolation of full-length *CmLOX10* and *CmLOX13*

Total RNA was extracted from the tender stem of melon using Ultrapure RNA Kit (Kangwei Biotech, China) and treated to eliminate genomic DNA using RNase-Free DNaseI (Takara, Japan). First-strand cDNAs were synthesized using M-MLV RTase cDNA Synthesis Kit (TaKaRa, Japan) following the manufacturer’s protocol and used for *CmLOX10* and *CmLOX13* cloning. Based on the *CmLOX10* and *CmLOX13* mRNA sequences from the melon genome database, PCR was performed with high-fidelity PrimeSTAR^™^ HS DNA Polymerase (Takara, Japan) in a 50 μl reaction mix containing 2 μl first-strand cDNA and 0.2 μM of each primer. The sense primer (CmLOX10-F and CmLOX13-F) and the antisense primer (CmLOX10-R and CmLOX13-R) were designed based upon the *CmLOX10* and *CmLOX13* sequences from the melon genome database and used for the cloning of the two full-length LOX genes. The PCR amplification conditions were as follow: 30 cycles of 10 s at 98°C, 15 s at 54°C for *CmLOX10* and 52°C for *CmLOX13* respectively, and 3 min at 72°C; 10 min at 72°C. The amplified about 3.0 kb LOX fragments were purified using TaKaRa MiniBEST Agarose Gel DNA Extraction Kit (Takara, Japan) according to the manufacturer’s protocols and inserted by TA-cloning into the pMD18-T vector (Takara, Japan) following adding A-tailing. The plasmids showing inserts of the expected size following plasmid PCR were sequenced by invitrogen (shanghai, China) and positive clones were designated as p18T-LOX10 and p18T-LOX13, respectively.

### PCR amplification of the 5’-end of *CmLOX10* and *CmLOX13*

To identify whether the coding sequences of *CmLOX10* and *CmLOX13* from the melon genome database acquired by RT-PCR were truly full-length, 5’ rapid amplification of cDNA ends (RACE) was performed using a commercial kit (Invitrogen, USA). Following the manufacturer’s recommendations, 5’ RACE of the *CmLOX10* was performed with the three gene-specific primers: LOX10-GSP1, LOX10-GSP2 and LOX10-GSP3, while 5’ RACE of the *CmLOX13* used LOX13-GSP1, LOX13-GSP2 and LOX13-GSP3.

### Bioinformatics analysis

The full-length *CmLOX10* and *CmLOX13* cDNAs acquired by a combination of RT-PCR and 5’ RACE-PCR were analyzed using several bioinformatics software. The calculated molecular mass and conserved domains of the deduced amino acid sequences of *CmLOX10* and *CmLOX13* were determined by ProtParam (http://web.expasy.org/protparam/) and the NCBI Conserved Domain Search program (http://www.ncbi.nlm.nih.gov/Structure/cdd/wrpsb.cgi), respectively. The deduced protein sequences of *CmLOX10* and *CmLOX13* were aligned using ClustalW2 software and displayed with GeneDoc. ClustalW2 software was used to align the deduced protein sequences of CmLOX10 and CmLOX13 with the protein sequences of other plant LOXs identified. The phylogenetic tree was then constructed using MEGA 5.0 based on the neighbor-joining method and a bootstrap value was calculated from 1000 replicates. The corresponding protein accession numbers of the identified LOX genes were acquired from NCBI GenBank (http://www.ncbi.nlm.nih.gov/Genbank). Subcellular localization was predicted by three different programs: WoLFPSORT (http://wolfpsort.org/), TargetP (http://www.cbs.dtu.dk/services/TargetP/), and ChloroP (http://www.cbs.dtu.dk/services/ChloroP/).

### Construction of *CmLOX10* and *CmLOX13* expression vectors

The DNA fragments of *CmLOX10* and *CmLOX13* containing *Eco*RI and *Xho*I restriction site were acquired using four PCR primers and two PCR reactions respectively according to the method described in the book Purifying Proteins for Proteomics: A Laboratory Manual [28]. Based on the predictions generated from the bioinformatics analysis, PCR primers were designed to amplify full-length and truncated sequences with putative transit peptide sequences removed. Using the p18T-LOX13 plasmid as template DNA, the truncated *CmLOX13* was amplified by the first pair of primers containing LOX13t-F-1 and LOX13t-R-1 and the second pair of primers containing LOX13t-F-2 and LOX13t-R-2 respectively to eventually generate two different PCR fragments of truncated *CmLOX13*. Similarly, the two different PCR fragments of the truncated *CmLOX10* were acquired by the primers including LOX10t-F-1, LOX10t-R-1, LOX10t-F-2 and LOX10t-R-2 and the full-length CmLOX10 were acquired by the primers containing LOX10f-F-1, LOX10f-R-1, LOX10f-F-2 and LOX10f-R-2 using the p18T-LOX10 plasmid as template DNA. Typical PCR reactions were performed with high-fidelity PrimeSTAR^™^ HS DNA Polymerase (Takara, Japan) according to the manufacturer’s instructions and the PCR condition was as similar as that described above. The PCR products were purified using TaKaRa MiniBEST Agarose Gel DNA Extraction Kit (Takara, Japan) according to the manufacturer’s protocols.

The same amount of two different purified PCR fragments of the truncated *CmLOX13* acquired above were mixed in a 50 μl reaction mixture containing 1 μl T4 polynucleotide kinase (Takara, Japan) and 1 μl 100 mM ATP (Takara, Japan) and the reaction conditions were as follow: 90 min at 37°C, 5 min at 95°C and 10 min at 25°C. The mixture containing the DNA fragments of truncated *CmLOX13* was directly ligated into the expression vector pET-30a (+) (Novagen, USA) digested by *Eco*RI and *Xho*I (Takara, Japan) using T4 DNA ligase (Takara, Japan) to generate pETt-CmLOX13 which encodes CmLOX13 with a His tag fused to the N terminus. The truncated and full-length *CmLOX10* expression vectors were constructed using the same method above designated as pETt-CmLOX10 and pET-CmLOX10, respectively. The truncated *CmLOX13* and the truncated and full-length *CmLOX10* ORF of these constructs were checked by sequencing prior to transform into *Escherichia coli* BL21 (DE3).

### Purification of recombinant CmLOX10 and CmLOX13 proteins

The transformants harboring pETt-CmLOX13, pETt-CmLOX10 and pET-CmLOX10 plasmids were inoculated in 2 ml of Luria-Bertani medium containing 50 μg ml^−1^ kanamycin and grown overnight at 37°C. The overnight culture of the *E*. *coli* BL21 (DE3) was transferred into one liter of LB medium containing the same amount of kanamycin and grown at 37°C until its optical density at 600 nm (OD600) reached approximately 0.6 followed by adding the isopropyl β-D-1-thiogalactopyranoside (IPTG) to a final concentration of 0.5 mM to induce the expression of the target proteins. The culture was subsequently incubated at 20°C for 18 h. Bacteria were harvested by centrifugation at 7000 rpm for 10 min at 4°C and the pellet was then resuspended in 40 ml of ice-cold lysis buffer (Kangwei Biotech, China) followed by lysing cell using sonication (5 s bursts and 7 s breaks for 30min on ice at 30% power) SONICATOR JY99-IIDN (Ningbo Scientz, China). After sonication on ice, the resulting slurry was centrifuged at 10000 g for 30 min at 4°C to pellet cell debris. The supernatant (soluble protein fraction) obtained as described above was filtered through a 45-μm filter and mixed with the same volume binding buffer (20 mM Tris-HCl, 0.5 M NaCl, 50 mM imidazole, pH 7.9). Then the mixture above and 2 ml of nickel-nitrilotriacetate agarose resin (Ni-NTA; Novagen, USA) pre-equilibrated with binding buffer was mixed and vibrated at 4°C for 1 h followed by adding to a 12-ml purification column (Kangwei Biotech, China).

The column was washed with the binding buffer and the nonspecific bound proteins were eluted. His-tagged proteins were then eluted with the elution buffer (20 mM Tris-HCl, 0.5 M NaCl, 500 mM imidazole, pH 7.9). The purified CmLOX10 and CmLOX13 were subjected to 7.5% SDS-PAGE and stained with Coomassie Brilliant Blue to confirm purity. Fractions of 1.5 ml showing LOX activity and containing pure protein on SDS-PAGE were concentrated and exchanged into 30 mM sodium phosphate, 50 mM NaCl (pH 7.5) by using an Amicon Ultra-15 membrane with a molecular weight cutoff of 30,000 (Millipore, USA) as described by Palmieri-Thiers et al [12]. All purification procedures were performed at 4°C. Protein concentration was determined using TaKaRa bradford protein assay kit (Takara, Japan) following the manufacturer’s instructions with bovine serum albumin as a standard.

### SDS-polyacrylamide gel electrophoresis (SDS-PAGE) and immunoblotting

SDS-PAGE was performed using 7.5% polyacrylamide gels. The proteins were stained with Coomassie brilliant blue R-250 or transferred onto nitrocellulose using a Trans-Blot Turbo blotting System (Bio-rad, USA). Blots were blocked overnight using 5% nonfat milk powder and the membrane was incubated for 12 h at 4°C with the anti His-tag mouse monoclonal antibody (Kangwei Biotech, China) at 1:3000 in western antibody dilution buffer (Kangwei Biotech, China). Then the membrane was washed three times for 10 min in TBST (20 mM Tris, 200 mM NaCl, 0.1% Tween-20) followed by incubating 30 min with HRP conjugated goat anti-mouse antibody (Kangwei Biotech, China) at 1:10000 in western antibody dilution buffer and washing three times for 10 min in TBST. Finally the membrane pretreated using Pro-light HRP Chemiluminescent Kit (Tiangen, China) according to the manufacturer’s instructions was exposed using ChemiDoc imaging system (Bio-rad, USA) for 1 min to detect the proteins containing histidine-tag.

### Characterization of the recombinant CmLOX10 and CmLOX13 proteins

LOX activity was determined by continuously monitoring the formation of conjugated dienes at 234 nm. Linoleic acid and linolenic acid were purchased from Sigma-Aldrich (Shanghai, China). The substrates were prepared according to the method described by Gata et al [29].

To estimate the optimum pH of the two recombinant LOX proteins, the reaction was run across a pH range of 4.0–9.0 at 25°C. Reaction buffers were prepared with 0.5 pH unit increments as follows: 100 mM sodium acetate (pH 4.0–5.5), 100 mM sodium phosphate (pH 6.0–7.5) and 100 mM sodium borate (pH 8.0–9.0). The effect of temperature on the activity of the two recombinant LOX proteins was determined at the temperatures ranging from 20 to 50°C with 5°C unit increments at pH 5.0 for CmLOX10 and 5.5 for CmLOX13.

Enzymatic assays were performed at saturating oxygen concentration at 25°C. The standard reaction mixture (3 ml) consisted of 0.33 mM linoleic acid or linolenic acid in 100 mM reaction buffer and 5 μl purified enzyme. The control reaction was carried out using the same pH reaction buffer but using water instead of the enzyme. Enzymatic activity was measured according to the method described by Liu et al [11] with a few modifications. The increase of the hydroperoxidaton was monitored at 234 nm at 25°C in a spectrophotometer (Cary 50, Varian, USA) for 3 min and the slope of the initial linear part of the plot was used to calculate the LOX activity. The maximum activity was estimated to be 100%. The kinetic parameters of CmLOX10 and CmLOX13 for linoleic and linolenic acid were calculated using Prism 5.0 (GraphPad Software) over a range of substrate concentrations between 16.5 and 330 μM in the standard reaction condition. One unit of LOX activity is defined as the amount of enzyme catalysing the formation of 1 μmol of hydroperoxide per minute at 25°C. All of the determinations were repeated three times.

The identity of the reaction products of recombinant CmLOX10 and CmLOX13 proteins was determined according to the method described by Long et al [30]. The 9-HPOD and 13-HPOD isomers were analyzed using a Waters HPLC equipment. The HPLC columns and conditions were similar to those described by Liu and Han [11]. The absorbance at 234 nm was recorded. Standards of 9- and 13-HPOD were purchased from Larodan (Malm, Sweden).

### Subcellular localization analysis by transient expression of CmLOX10 and CmLOX13 in tobacco leaves

Two GFP fusion constructs were produced to investigate the subcellular localization of CmLOX10 and CmLOX13. Using the p18T-LOX10 and p18T-LOX13 plasmids as templates, the full-length *CmLOX10* and *CmLOX13* without stop codon were re-amplified by the primers containing LOX10G-F/R and LOX13G-F/R, respectively. Typical PCR reactions were performed with high-fidelity PrimeSTAR^™^ HS DNA Polymerase (Takara, Japan) according to the manufacturer’s instructions and the PCR conditions were as follow: 30 cycles (denaturation at 98°C for 10 s, annealing at 52°C for 15 s, extension at 72°C for 3 min, final extension at 72°C for 10 min). The PCR product was purified using TaKaRa MiniBEST Agarose Gel DNA Extraction Kit (Takara, Japan) according to the manufacturer’s protocols and cloned into a pENTR^™^/D-TOPO^®^ vector (Invitrogen, USA) followed by transforming into DH5α competent *E*. *coli* cells (Takara, Japan). Plasmids from five randomly selected clones for each gene were extracted, checked by restriction digest and sequenced by invitrogen (shanghai, China). The resulting entry vectors were used to deliver the *CmLOX10-GFP* and *CmLOX13-GFP* constructs into the plant expression vector pB7FWG2 [31] using Gateway^®^ LR Clonase^™^ II Enzyme Mix (Invitrogen, USA) according to the manufacturer’s instructions. After transformation and sequencing, the binary vectors which were designated as LOX10-GFP and LOX13-GFP were transferred into the *Agrobacterium tumefaciens* strain GV3101 by freeze-thaw transformation.

Transformant *Agrobacterium* harboring LOX10-GFP and LOX13-GFP binary vectors were inoculated in 5 ml of YEB medium containing 50 μg ml^−1^ rifampicin, 50 μg ml^−1^ spectinomycin, 10 mM ethanesulfonic acid (pH 5.7 MES) and 20 μM acetosyringone (AS) and grown overnight at 28°C, 200 rpm. Cells were harvested by centrifugation at 5000 rpm for 10 min at room temperature. After supernatant was removed, added 2 ml of infiltration media (10 mM pH 5.7 MES, 10 mM MgCl_2_, and 150 μM AS) and resuspend. Repeat the step above and diluted using the infiltration media to OD_600_ = 0.8. *Agrobacterium* suspensions harboring LOX10-GFP and LOX13-GFP binary vectors were injected into the abaxial surfaces of the leaves of the tobacco (*Nicotiana benthamiana*) grown for about 6 weeks using a 1ml needleless syringe. Then the tobacco plants were maintained in a growth cabinet under a 16/8 h day/night cycle at 25°C. The leaves of tobacco plants at 4 days after infiltration were collected to analyze the subcellular localization of CmLOX10 and CmLOX13 fusion proteins.

Both green fluorescent signals and spontaneous red fluorescent signals of chloroplasts were detected by confocal laser scanning microscopy (LSM510, Zeiss, Germany). GFP fluorescence was excited with the 488-nm Ar laser line and the emission signals were collected between 505 and 525 nm for GFP and between 640 and 720 nm for chlorophyll autofluorescence. All fluorescence experiments were repeated three times.

## Results

### *CmLOX10* and *CmLOX13* cDNAs isolation and characterization

We isolated two full-length cDNAs by a combined strategy using RT-PCR and RACE-PCR on total RNA extracted from the tender stem of oriental melon. The nucleotide and amino acid sequences of oriental melon CmLOX10 and CmLOX13 were analyzed by bioinformatics software (Table 1).

**Table 1:** Nucleotide sequence analysis of oriental melon *CmLOX10* and *CmLOX13* and predicted protein length, molecular mass and pI.

LOX name	ORF length (bp)	5’UTR length (bp)	3’UTR length (bp)	Predicted protein length (aa)	Predicted molecular mass (kDa)	pI
**CmLOX10**	2709	47	118	902	102.301	6.0
**CmLOX13**	2721	48	21	906	102.312	6.34

The *CmLOX10* (GenBank: KT613843) harbours a 2709-bp open reading frame (ORF) flanked by a 47 bp 5’-untranslated region (UTR) and 118 bp 3’-UTR. A TAA stop codon in the 5’- UTR of *CmLOX10* is found in 9 bp upstream from the first ATG which is the start codon of *CmLOX10*. The deduced CmLOX10 protein sequence consists of 902 amino acid residues with a calculated molecular mass of 102.301 kDa and a theoretical isoelectric point (pI) of 6.0. The *CmLOX13* (GenBank: KT613844) harbours a 2721-bp ORF encoding a predicted protein of 906 amino acid residues with a calculated molecular mass of 102.312 kDa and a theoretical pI of 6.34. The ORF of *CmLOX13* is flanked by the 5’-UTR of 48 bp and 3’-UTR of 21 bp.

Two sequence alignments of the nucleotides and deduced amino acid sequences of CmLOX10 isolated from oriental melon (*Cucumis melo* var. *makuwa* Makino) and from the melon (*Cucumis melon* L.) genome database (https://melonomics.net) were done and seven different nucleotides and three different amino acids are found (S1 Fig). For CmLOX13, there are five different nucleotides and one different amino acid (S2 Fig).

### Bioinformatics analysis of CmLOX10 and CmLOX13

**Fig 1:**
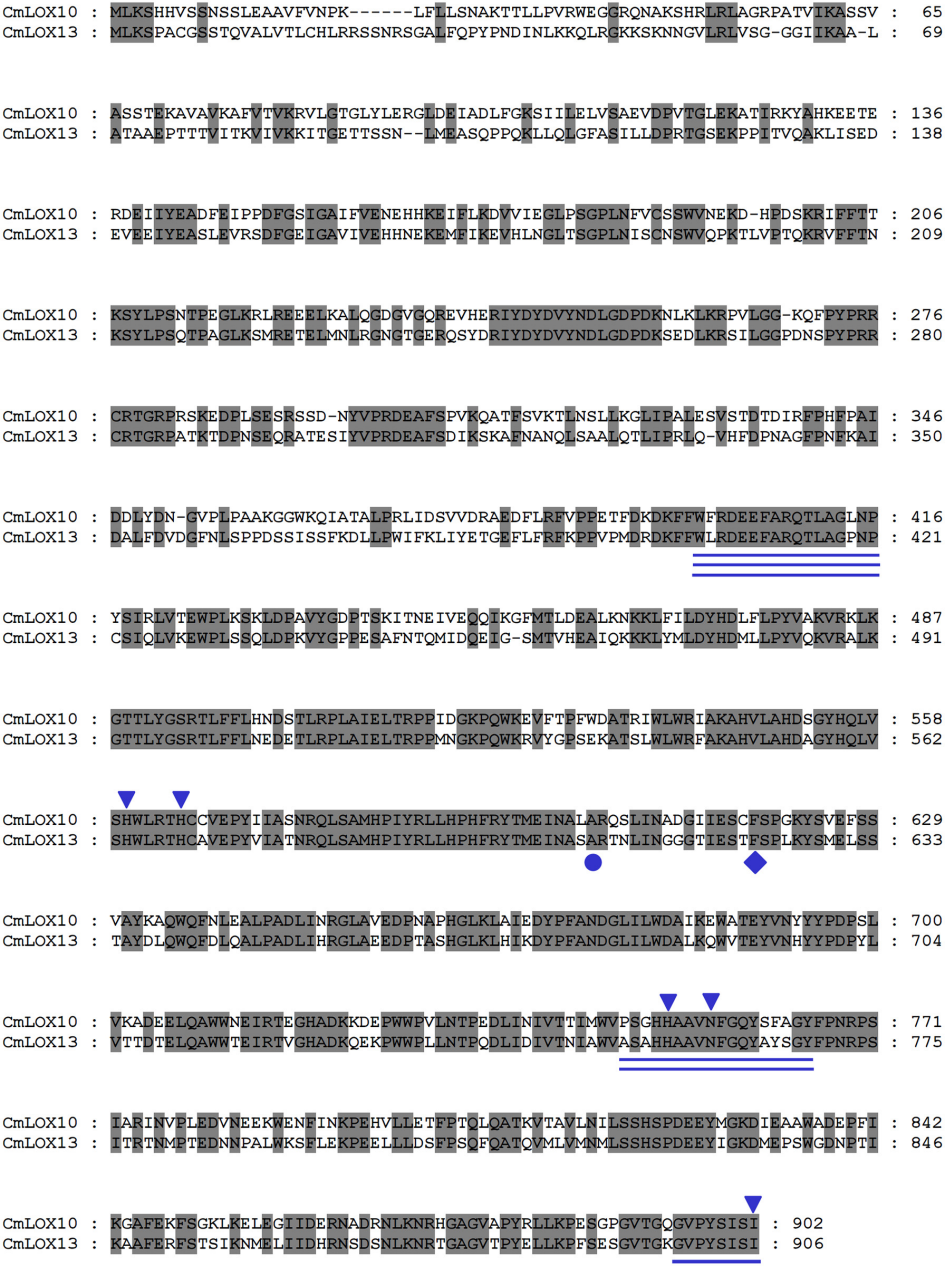
Alignment of the deduced amino acid sequences of oriental melon CmLOX10 and CmLOX13. The deduced amino acid sequences of two LOXs were aligned using ClustalW2 software and displayed with GeneDoc. The C-terminal conserved domain of lipoxygenases is single underlined. The conserved domains involved in oxygen binding and substrate binding are double and triple underlined, respectively. The conserved amino acids of the two LOXs which are three His, one Asn and one Ile residues that involve in iron binding and enzyme catalytic activity are indicated by inverted triangles. CmLOX10 and CmLOX13 proteins which possess the Phe residue denoted by a rhomb are predicted to be 13-LOXs. The Ala residue that determines the S-stereospecificity of most LOXs is denoted by a round.

By analyzing the deduced amino acid sequences of CmLOX10 and CmLOX13, the PLAT_LH2 and LOX domains of the deduced CmLOX10 protein which are the two conserved domains of plant LOXs and located in 73–205 and 215–885 amino acids respectively were identified, while the PLAT and LOX domains of the deduced CmLOX13 protein are located in 108–209 and 218–890 amino acids respectively (S3 Fig). Sequence alignments of the predicted amino acid sequences of the CmLOX10 and CmLOX13 showed that they shared 58.11% identity with each other (Fig 1). Several highly conserved regions of the predicted CmLOX10 and CmLOX13 proteins were identified by amino acid sequence alignments. As shown in Fig 1, the conserved domains involved in substrate binding and oxygen binding, highly conserved C-terminal motif and the conserved amino acids of the two LOXs which are three His, one Asn and one Ile residues that involve in iron binding and enzyme catalytic activity are found in predicted CmLOX10 and CmLOX13 proteins [32]. Furthermore, CmLOX10 and CmLOX13 possess the conserved Cys/Phe motif (C_617_ and F_618_) and Thr/Phe motif (T_621_ and F_622_) respectively and are predicted to be 13-LOXs. The conserved Ala residue which determines the S-stereospecificity of most LOXs is also observed [33].

**Fig 2:**
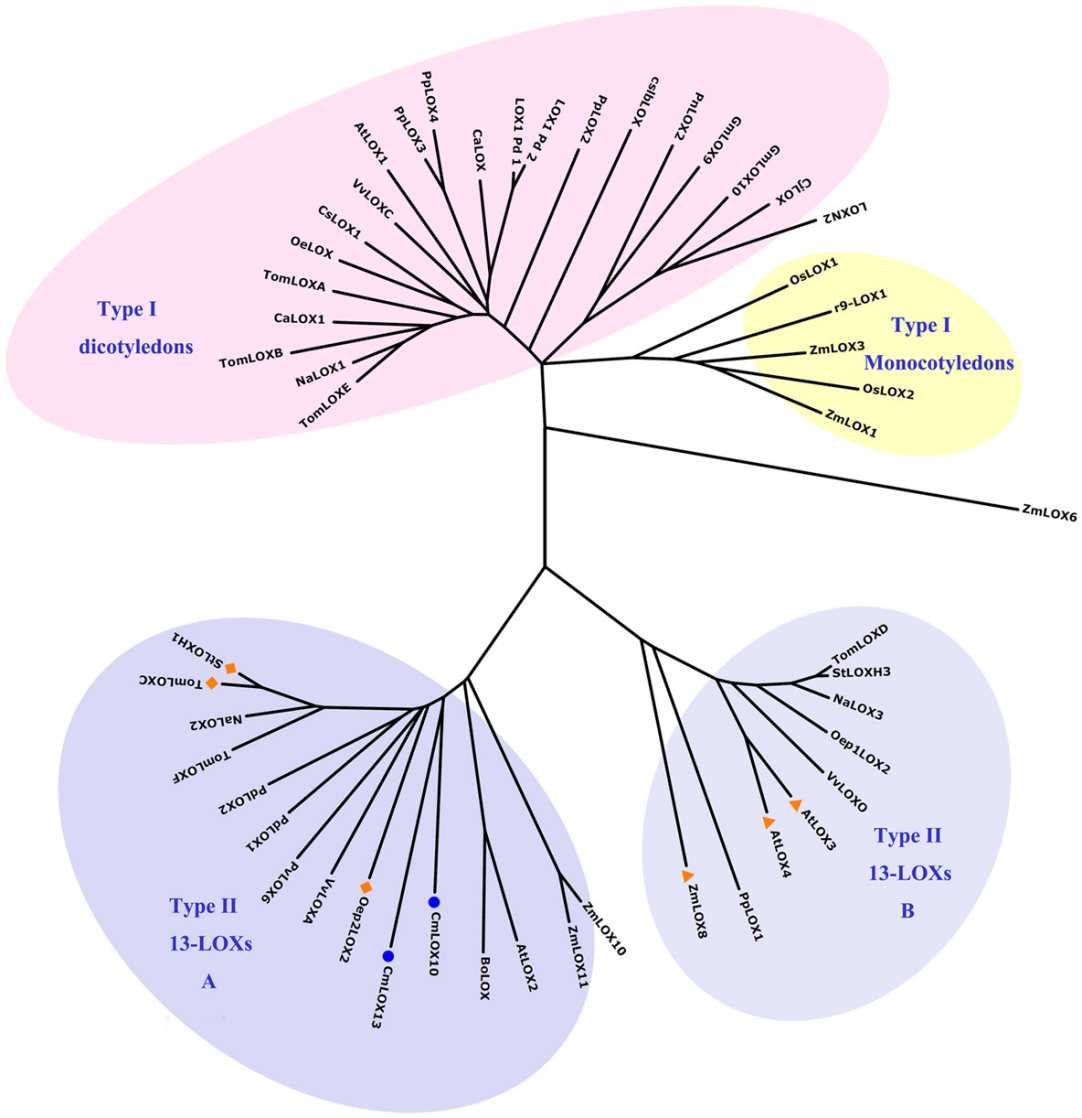
Phylogenetic analysis of the deduced amino acid sequences of CmLOX10, CmLOX13 and other biochemically characterized plant LOXs. The deduced amino acid sequences were aligned by the ClustalW2 software. The phylogenetic tree was constructed using MEGA 5.0 software based on the neighbor-joining method and a bootstrap value was calculated from 1000 replicates. The shaded areas outline four separate LOX classes. CmLOX10, CmLOX13 isolated in this work are indicated by a round. StLOXH1, TomLOXC and Oep2LOX2 in the type II A subgroup which play a key role in the generation of fatty-acid-derived short-chain volatiles are indicated by a rhomb. AtLOX3, AtLOX4 and ZmLOX8 in the type II B subgroup which maybe involve in the biosynthesis of the plant hormone jasmonic acid and further influences flower development are indicated by a triangle. Accession numbers of the different lipoxygenases included in the analysis: *Arabidopsis thaliana* (AtLOX1, NP_175900; AtLOX2, AAL32689; AtLOX3, CAB56692; AtLOX4, NP_177396); *Arachis hypogaea* (PnLOX2, AAY87056); *Brassica oleracea* (BoLOX, ABO32545); *Capsicum annuum* (CaLOX1, ACO57136); *Caragana jubata* (CjLOX, ABQ10187); *Camellia sinensis* (CsLOX1, ABW75772); *Corylus avellana* (CaLOX, CAD10740); *Cucumis sativus* (cslbLOX, CAA63483); *Glycine max* (GmLOX9, ABS32275; GmLOX10, ABS32276); *Lycopersicum esculentum* (TomLOXA, AAA53184; TomLOXB, AAA53183; TomLOXC, AAB65766; TomLOXD, AAB65767; TomLOXE, AAG21691; TomLOXF, ACM77790); *Nicotiana attenuata* (NaLOX2, AAP83137; NaLOX3, AAP83138); *Nicotiana tabacum* (NaLOX1, CAA58859); *Olea europaea* (OeLOX, ACG56281; Oep1LOX2, ACD43484; Oep2LOX2, ACD43485); *Oryza sativa* (OsLOX1, ABD47523; OsLOX2, CAA45738; r9-LOX1, BAD02945); *Phaseolus vulgaris* (PvLOX6, ABM88259); *Pisum sativum* (LOXN2, CAG44502); *Populus deltoids* (PdLOX1, AAZ57444; PdLOX2, AAZ57445); *Prunus dulcis* (LOX1:Pd:1, CAB94852; LOX1:Pd:2, CAD10779); *Prunus persica* (PpLOX1, ACG59769; PpLOX2, ACH90245; PpLOX3, ACH91370; PpLOX4, ABV32552); *Solanum tuberosum* (StLOXH1, CAA65268; StLOXH3, CAA65269); *Vitis vinifera* (VvLOXA, ACZ17391; VvLOXC, ACZ17392; VvLOXO, ACZ17393); *Zea mays* (ZmLOX1, AAF76207; ZmLOX3, AAG61118; ZmLOX6, ABC59689; ZmLOX8, ABC59691; ZmLOX10, ABC59693; ZmLOX11, ABC59694).

To predict the regiospecificity and potential subcellular location of the CmLOX10 and CmLOX13, multiple sequence alignments of the putative amino acid sequences of the two oriental melon LOXs and other plant LOXs of which the functions were characterized were calculated with ClustalW2 software and a phylogenetic tree was constructed using MEGA 5.0 software (Fig 2). According to the classification of Feussner and Wasternack [1], the tree could be divided into type I and type II lipoxygenases. Type I lipoxygenases which lack plastid targeting peptides and share over 75% sequence similarity among themselves can be further divided into monocotyledons LOXs and dicotyledons LOXs. Monocotyledons LOXs in the phylogenetic tree are linked to defense against pests and pathogen attack, while dicotyledons LOXs play a role in plant growth and development and adversity stress. Type II lipoxygenases which carry a plastid-targeting peptide and share low sequence similarity with members of the subgroup can be further divided into A and B subgroups which are related to mechanical damage and pathogen infection. AtLOX3, AtLOX4 and ZmLOX8 (tasselseed1) enzymes in the B subgroup maybe involve in the biosynthesis of the plant hormone jasmonic acid and further influences flower development [19,34], while StLOXH1, TomLOXC and Oep2LOX2 in the A subgroup play a key role in the generation of fatty-acid-derived short-chain volatiles [18,35,36]. The phylogenetic tree clearly shows that CmLOX10 and CmLOX13 belong to the A subgroup of type II LOX group. Furthermore, it is predicted that the deduced CmLOX10 and CmLOX13 proteins may possess transit peptides for chloroplast targeting by means of three different programs which are TargetP1.1, WoLFPSORT, and ChloroP1.1. On the basis of the prediction of TargetP1.1and ChloroP1.1 programs, the length of the chloroplast transit peptide of the N- terminus of the deduced CmLOX10 and CmLOX13 proteins are 37 and 23 respectively and the two proteins likely encode chloroplast-targeted LOXs.

### Purification of the recombinant CmLOX10 and CmLOX13

**Fig 3:**
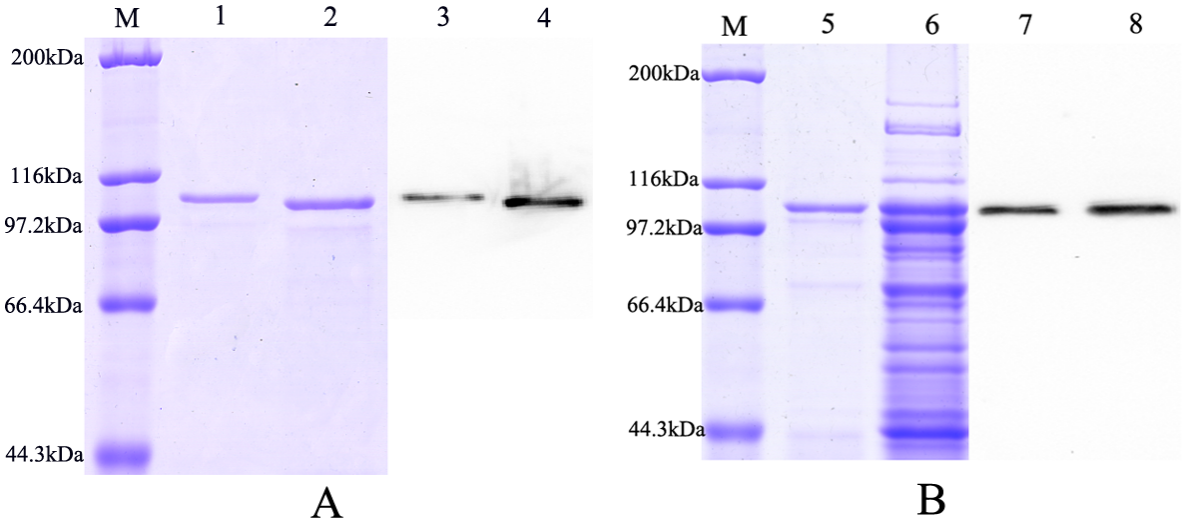
SDS–PAGE and Western blotting analysis of the purified His-tagged truncated CmLOX13 and CmLOX10 and full-length CmLOX10 recombinant proteins. Lane M: Protein Molecular Weight Marker. (A) SDS-PAGE of purified His-tagged truncated CmLOX13 and CmLOX10 recombinant proteins (lane 1 and 2) and were detected on Western blotting with an anti-His-tag antibody (lane 3 and 4). (B) SDS-PAGE of purified His-tagged full-length CmLOX10 recombinant protein and soluble fraction of *E*. *coli* BL21(DE3) cell extracts expressing His-tagged CmLOX10 protein (lane 5 and 6) and were detected on Western blotting with an anti-His-tag antibody (lane 7 and 8).

It showed that CmLOX10 and CmLOX13 proteins were likely located in chloroplast by analyzing the CmLOX10 and CmLOX13 sequence using the TargetP1.1 and ChloroP1.1 bioinformatics programs. As the chloroplast transit peptide can provoke some problems during the production in bacteria [15,37], we cloned and expressed the CmLOX10 and CmLOX13 cDNAs in *E*. *coli*, without their chloroplast transit peptide, but with a poly-His tag. Total proteins were extracted by sonication and the soluble fusion proteins were obtained in the supernatant and purified by affinity chromatography. The purity of the purified recombinant CmLOX10 and CmLOX13 proteins were checked by SDS-PAGE and Western blotting with an anti-His-tag antibody. SDS–PAGE and Western blotting analysis (Fig 3A) revealed two unique bands which were in good agreement with the predicted molecular mass of CmLOX10 (103.97 kDa, including the His-tag) and CmLOX13 (105.56 kDa, including the His-tag).

Unfortunately, we could not detect LOX activity of truncated recombinant CmLOX10, so we expressed the full-length of CmLOX10 including the chloroplast transit peptide in *E*.*coli* as a fusion protein. After induction of expression and purifying, purified recombinant CmLOX10 expressed in *E*. *coli* was detected on Coomassie Brilliant Blue-stained SDS-PAGE gels and by Western blotting with an anti-His-tag antibody. SDS-PAGE analysis revealed that one band which was similar to the predicted molecular mass of the CmLOX10 (108.01 kDa, including the His-tag) was detected in both soluble supernatant fractions of the cells and purified recombinant CmLOX10 (Fig 3B).

### Biochemical characterization of the full-length recombinant CmLOX10 and truncated CmLOX13

**Fig 4:**
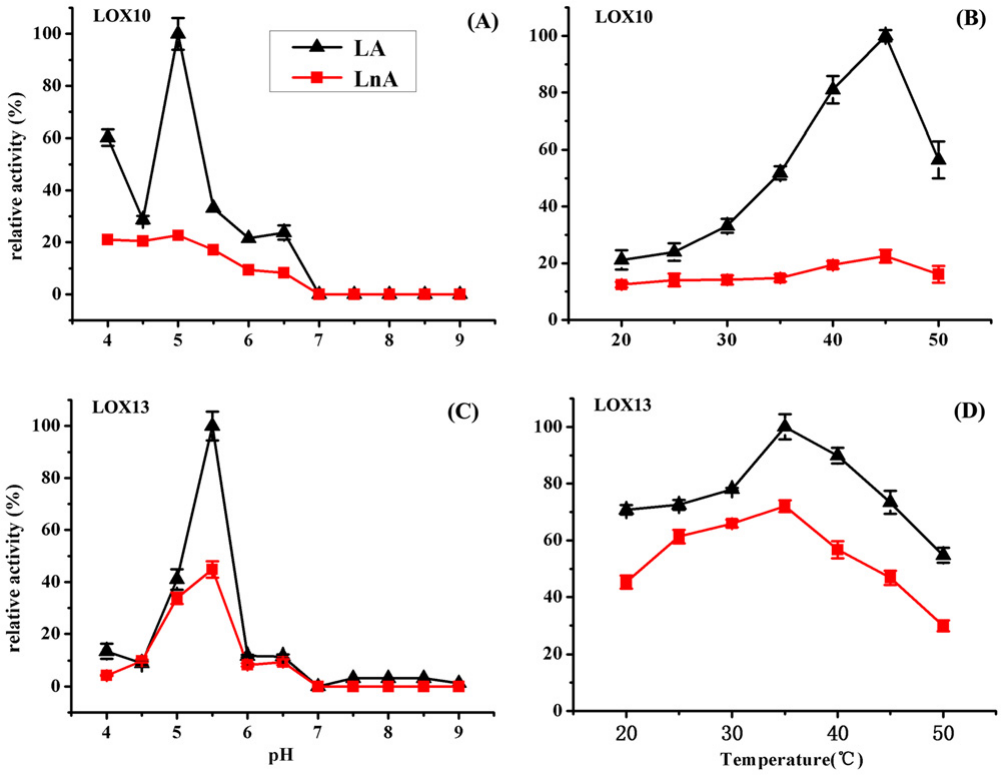
The effects of pH and temperature on the enzymatic activity of recombinant CmLOX10 (A, B respectively) and CmLOX13 (C, D respectively) were determined using linoleic acid (LA) and linolenic acid (LnA) as substrates. The recombinant CmLOX10 and CmLOX13 proteins displayed the highest catalytic activity at pH 5.0 (A) and 5.5 (C) respectively. The optimum temperatures for CmLOX10 and CmLOX13 were observed at 45°C (B) and 35°C (D), respectively. The maximum activity was estimated as 100%. Means ± SD were obtained from three independent measurements.

To determine the optimal pH for full-length recombinant CmLOX10 and truncated recombinant CmLOX13, the enzyme activity was analyzed at 25°C and different pH values (pH 4.0–9.0), using linoleic and linolenic acids as substrates which are the two major PUFAs in plants. As shown in Fig 4, the recombinant CmLOX10 and CmLOX13 proteins displayed the highest catalytic activity at pH 5.0 and 5.5 respectively and activities dramatically decreased when pH was higher than 6.0. To determine the optimum temperature for both recombinant LOXs, enzyme activity was measured over a range of temperatures (20–50°C). The optimum temperature for CmLOX10 and CmLOX13 were observed at 45°C and 35°C, respectively. The enzyme activity of CmLOX10 increased as temperature rise between 20 and 45°C and then decreased at 50°C. In comparison, the enzyme activity of CmLOX13 was sharply decreased when the temperature was higher than 35°C (Fig 4B and 4D).

**Fig 5:**
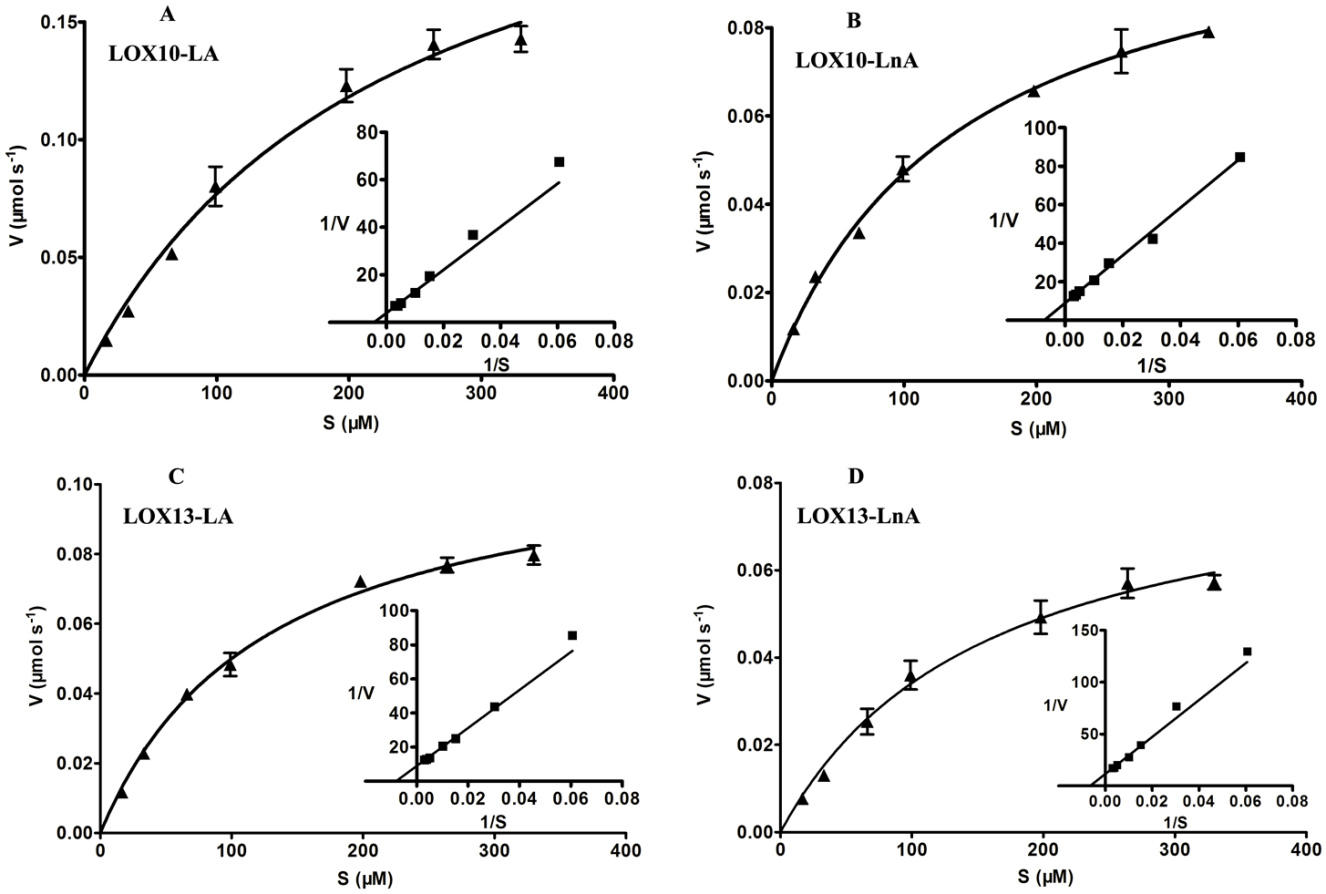
Recombinant CmLOX10 (A, B) and CmLOX13 (C, D) kinetic constants were determined using linoleic acid (LA) and linolenic acid (LnA) as substrates. Values represent the means ± SD of three independent replicates. Inset represent Lineweaver-Burk plot.

To determine the kinetic characteristics of the recombinant CmLOX10 and CmLOX13 proteins, the enzyme activity was assayed by monitoring the formation of hydroperoxides over a range of concentrations of the main substrates (linoleic and linolenic acid) between 16.5 and 330 μM using standard conditions. Furthermore, the kinetic parameters using linoleic and linolenic acid as substrates were calculated by a Michaelis-Menten plot analysis program (Fig 5). The kinetic parameters of the recombinant CmLOX10 and CmLOX13 are summarized in Table 2. The comparison of the *V*_max_ values indicats that CmLOX10 and CmLOX13 oxidize linoleic acid about 2.3 and 1.3-fold more quickly than linolenic acid, respectively. The recombinant CmLOX10 and CmLOX13 show a higher *k*_cat_/*K*_m_ values for linoleic acid than for linolenic acid, suggesting that CmLOX10 and CmLOX13 have the highest catalytic efficiency with linoleic acid. These results above indicate that linoleic acid is the preferred substrate for the recombinant CmLOX10 and CmLOX13.

**Table 2:** Kinetic parameters of purified recombinant CmLOX10 and CmLOX13.

Enzyme	Substrate	*K*_m_ (μM)	*V*_max_ (μmol s^-1^)	*k*_cat_ (s^-1^)	*k*_cat_/*K*_m_ (s^-1^μM^-1^)
**CmLOX10**	**Linoleic acid**	230.3	0.2543	5492.88	23.85
	**Linolenic acid**	140.2	0.1131	2442.96	17.42
**CmLOX13**	**Linoleic acid**	125.8	0.1129	2438.64	19.39
	**Linolenic acid**	156.6	0.0877	1841.7	11.76

**Fig 6:**
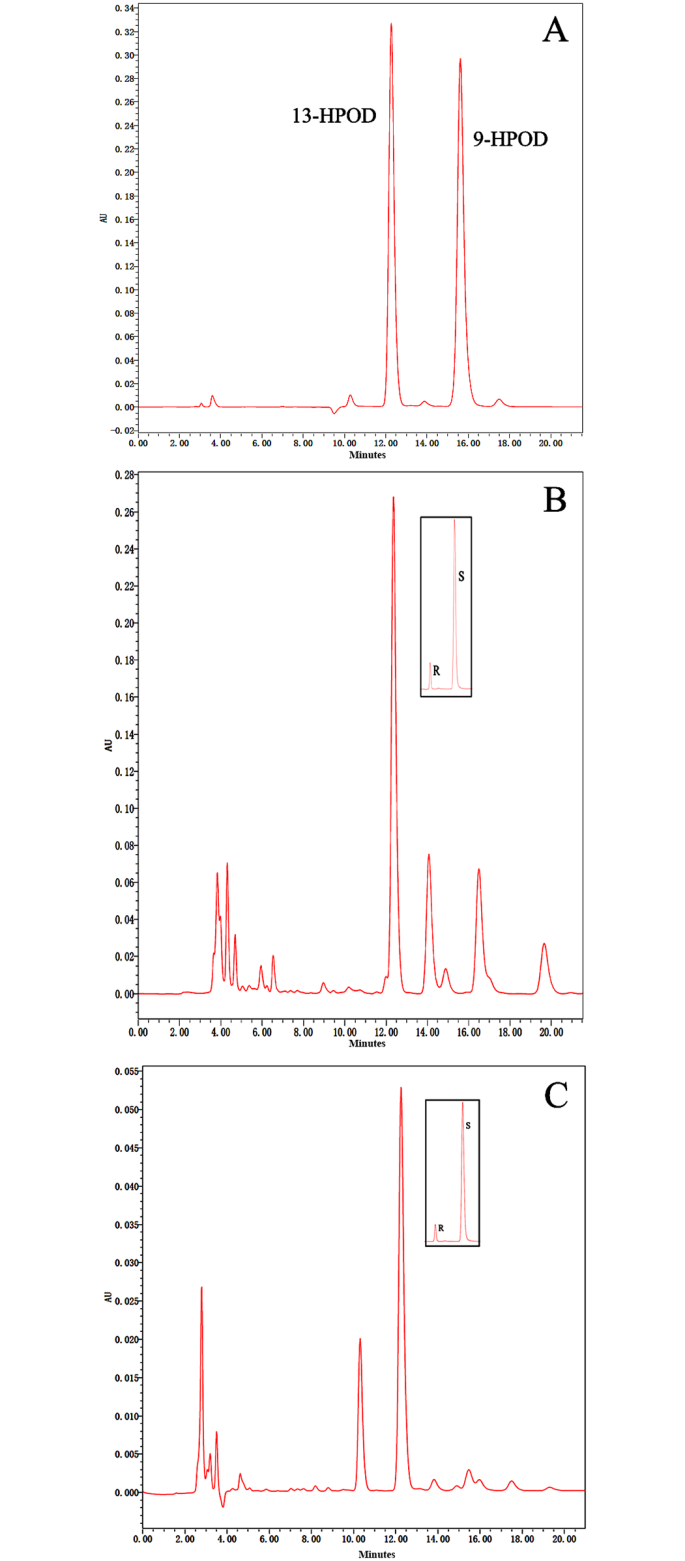
Determination of positional specificity of the recombinant CmLOX10 and CmLOX13. A shows the retention time of the isomers produced by soybean LOX1. B and C: SP-HPLC analysis of the reaction products of recombinant CmLOX10 and CmLOX13. Boxes: chiral-phase HPLC showing the enantiomer composition of 9- and 13-HPOD.

To determine the positional specificity of purified CmLOX10 and CmLOX13, we further identified the reaction products from the CmLOX10 and CmLOX13 enzyme reaction following the SP-HPLC analysis. As shown in Fig 6, 13-hydroperoxyoctadecadienoic acid (13-HPOD) was mainly produced by the reaction of purified CmLOX10 and CmLOX13 with linoleic acid. The retention time of the reaction products was consistent with authentic 13-HPOD standards, indicating that CmLOX10 and CmLOX13 were 13-LOXs. In addition, 13-HPOD was predominantly in the S configuration by Chiral-phase HPLC analysis of the reaction products, indicating that that they derived from the activity of a specific enzyme. The results above reveal that CmLOX10 and CmLOX13 are 13S-LOX enzymes and convert linoleic acid into 13(S)-hydroperoxyoctadecadienoic acid (13(S)-HPOD).

### Subcellular localization of CmLOX10 and CmLOX13 proteins

**Fig 7:**
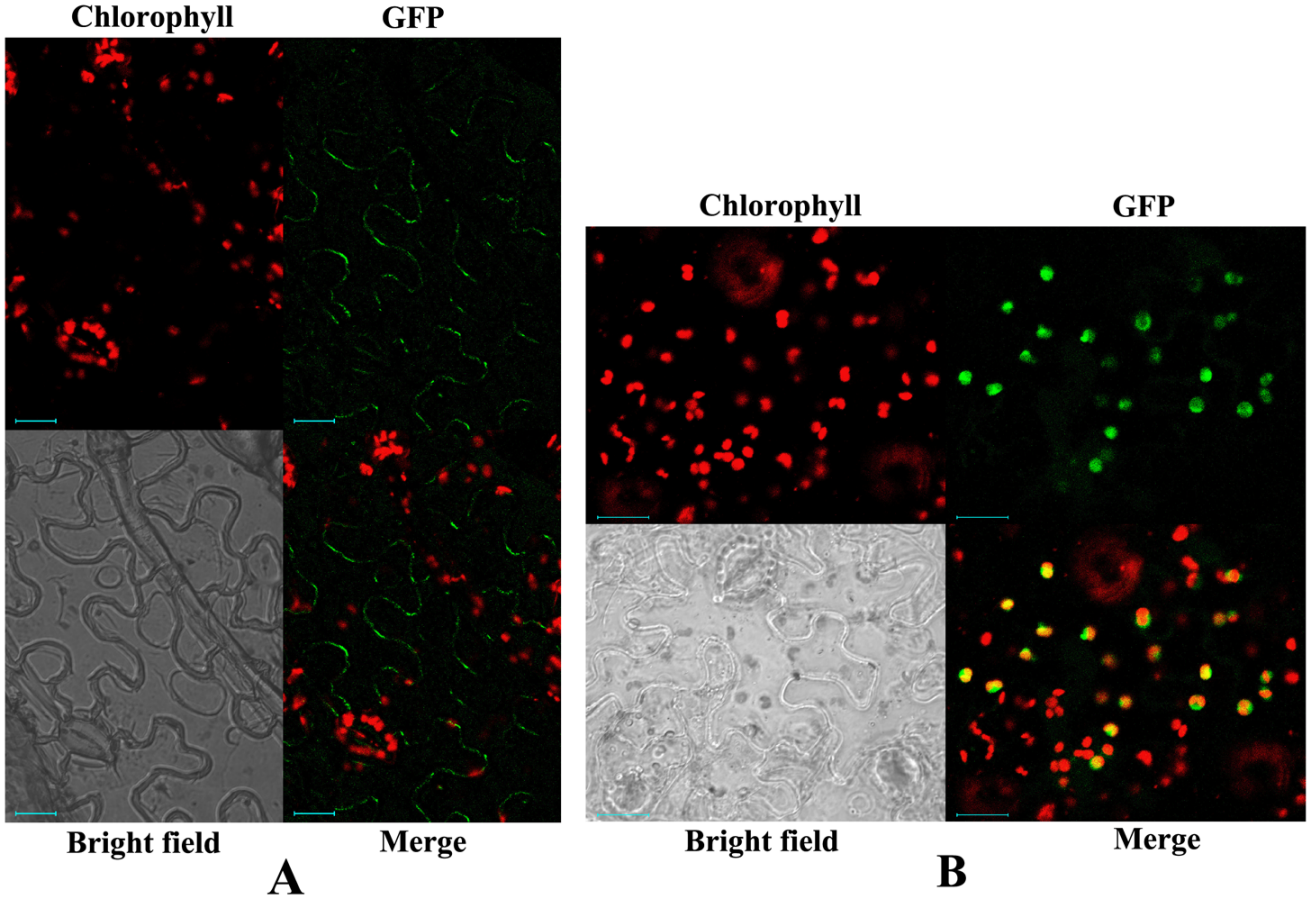
Subcellular localization analysis by transient expression of CmLOX10 and CmLOX13 fusion constructs in tobacco leaves. Tobacco leaves were infiltrated with *Agrobacterium tumefaciens* carrying the LOX10-GFP (A) and LOX13-GFP constructs (B). Both green fluorescent signals and spontaneous red fluorescent signals of chloroplasts were detected using Zeiss LSM510 confocal microscope excited with a 488-nm Ar laser line. Merge is the computed overlay of the two fluorescence images. Reference bar is 20 μm.

In order to determine the in vivo localization of CmLOX10 and CmLOX13, we transiently expressed CmLOX10-GFP and CmLOX13-GFP fusion constructs in tobacco leaves under the control of the cauliflower mosaic virus 35S promoter. We examined the CmLOX10-GFP and CmLOX13-GFP fusion protein localization using confocal laser scanning microscopy. As shown in Fig 7, CmLOX10 protein was located in plasma membrane, while CmLOX13-GFP fluorescence signals co-localized with the auto-fluorescence of chlorophyll, indicating that the subcellular localization of CmLOX10 protein was not in accord with the result of the bioinformatics analysis and CmLOX13 protein was located in chloroplasts. The results above show that CmLOX10 and CmLOX13 have different locations and may play different functions during plant growth and development, although they have a high degree of identity at the amino acids level with each other.

## Discussion

Using RT-PCR and RACE-PCR combined approach, the full length of *CmLOX10* and *CmLOX13* which were isolated from the tender stem of oriental melon cultivar “Yumeiren” grown for about 30 days were cloned into T vector and sequenced. Comparing the sequencing results with the sequences of *CmLOX10* and *CmLOX13* from melon genome database, a few different nucleotides and deduced amino acids were found. The differences above should due to varieties difference. The oriental melon cultivar “Yumeiren” is used in our experiment, while the melon doubled-haploid line DHL92 is used in genome sequence [26].

In plants, which type LOXs are classified into is based on the positions where the oxygenation of linoleic acid occurs. Generally, there are some conservative sites in plant LOXs that are related with the positional specificity and stereo-specificity of LOXs. A space-filling His or Phe residue at the bottom of the substrate-binding pocket was identified in nearly all plant 13-LOXs, while a Val residue was identified in plant 9-LOXs [6,38]. For stereo-specificity of LOXs, the conservative residue is Ala for S-LOX and Gly for R-LOX [39]. However, it has been shown that some plant LOXs could produce 9- and13-hydroperoxides by the heterologous expression method, such as Pea (*Pisum sativum*) LOXN2, olive (*Olea europaea* L.) LOX and tea plant (*Camellia sinensis*) CsLOX1 [11,12,16]. By analyzing the deduced CmLOX10 and CmLOX13 amino acid sequences, the two proteins include all the domains involved in substrate, oxygen binding, the binding of the atom of iron and Phe and Ala residues which are related with the positional specificity and stereo-specificity of LOXs and are predicted to be 13S-lipoxygenases. It has been proven by the analysis of product specificity using HPLC. Our results provide new information for the relationship between product specificity and the amino acids sequence.

The intracellular localization of LOXs may provide some predictions about the physiological functions of the different LOXs [1]. Besides cytoplasm and plastids, LOXs have been found in many other organelles, such as the vacuole, peroxysomes, lipid bodies, plasma membranes and microsomal membranes [2]. Furthermore, ZmLOX6 protein had been isolated from mesophyll cell chloroplasts, although bioinformatics analysis suggested the protein was located in cytoplasm [23]. The prediction of subcellular localization of the CmLOX10 and CmLOX13 proteins was done by means of three different programs and the bioinformatics analysis showed that the length of the chloroplast transit peptide of CmLOX10 and CmLOX13 were 37 and 23, respectively. However, subcellular localization analysis by transient expression of CmLOX10 and CmLOX13 in tobacco leaves showed that CmLOX10 and CmLOX13 were located in plasma membrane and chloroplasts, respectively. It is possible that the different LOXs may have a specific location and play different roles in plant growth and development.

It might be helpful in predicting biochemical features and physiological functions of the LOXs by phylogenetic tree analysis [1], so the phylogenetic tree including CmLOX10, CmLOX13 and other plant LOXs of which the functions have been characterized was constructed. Our phylogenetic analysis shows that oriental melon CmLOX10 and CmLOX13 belong to the type II 13-LOXs in which all the LOX proteins identified are 13-LOXs and known to involve in biotic and abiotic stresses and are classified in the A subgroup including StLOXH1, TomLOXC and Oep2LOX2 which all involve in the generation of fatty-acid-derived short-chain volatiles.

Expression of the truncated CmLOX10 protein with putative transit peptide sequences removed failed to detect LOX activity, so we produced full-length His-tagged CmLOX10 expression vectors and expressed in *E*. *coli*. Chromatogram analysis of reaction products revealed that the full-length CmLOX10 and truncated CmLOX13 possessed the predicted 13-LOX activity and produced 13(S)-HPODE using linoleic acid as substrate. Although producing the same products, CmLOX10 and CmLOX13 exhibited different kinetic properties and optimal reaction conditions (Table 2 and Fig 4). CmLOX10 had the maximal activity when the pH and temperature were 5.0 and 45°C respectively, while CmLOX13 exhibited the highest catalytic rates at pH 5.5 and 35°C. Linoleic and linolenic acid which are the most common plant substrates for LOXs [40] were used as the substrates in the analysis of the enzymatic activity of recombinant CmLOX10 and CmLOX13. The catalytic efficiency of CmLOX10 and CmLOX13 (*k*_cat_/*K*_m_) was higher with linoleic acid than with linolenic acid, so both CmLOX10 and CmLOX13 recombinant enzymes exhibited a preference for linoleic acid.

The metabolism of PUFAs via the LOX-catalyzed step and the subsequent reactions are collectively named the LOX pathway [1]. The LOX pathway has several branches and produces many signaling molecules commonly by activating the allanene oxide synthase (AOS) or hydroperoxide lyase (HPL) branches. 13-hydroperoxylinolenic acid produced by 13-LOX is converted into jasmonate via AOS branch also called octadecanoid pathway, whereas HPL leads to the formation of ω-oxo acids and volatile C6 and C9 aldehydes followed by metabolizing by alcohol dehydrogenase (ADH) into the corresponding alcohols in HPL branch [1,41]. Although the formation of JA and Green Leaf Volatiles (GLVs) which are C6 or C9-aldehydes and alcohols utilize hydroperoxides as a common substrate, it have been demonstrated that LOX isoforms could provide substrates for one of the AOS and HPL branches. For example, the main role of potato StLOXH1 is to supply substrates for the HPL branch to produce C6 volatiles, but not to supply hydroperoxides for the AOS branch [42]. In contrast, NaLOX3 in *Nicotiana attenuate* and rice OsHI-LOX supply substrates for the AOS branch but not the HPL branch [43,44]. In maize, ZmLOX10 and ZmLOX8 specialize in providing substrates for the production of green leaf volatile (GLV) and the biosynthesis of jasmonate (JA), respectively [45]. Furthermore, whether the LOX is involved in the production of GLV or the biosynthesis of JA is determined by the positional specificity of the LOX, so we analyzed the reaction products by HPLC analysis for the positional specificity of CmLOX10 and CmLOX13. The result showed that 13S-HPOD was produced by the reaction of purified CmLOX10 and CmLOX13 with linoleic acid. In consideration of the phylogenetic tree analysis of the two LOXs which belong to the subgroup including StLOXH1, TomLOXC and Oep2LOX2 which all involve in the generation of fatty-acid-derived short-chain volatiles, it can be predicted that CmLOX10 and CmLOX13 are likely to participate in the formation of C6 volatiles which play roles both in development and the defense response in plants [46,47].

We have previously shown that CmLOX10 and CmLOX13 expressed in different tissues of oriental melon cultivar “Yumeiren” in growth and development. *CmLOX10* expressed during fruit ripening, while the expression of *CmLOX13* mRNA appeared at earlier stage of fruit ripening (S4 Fig). To understand the roles of *CmLOX10* and *CmLOX13* in defense responses, the expressions of the two genes in response to mechanical wounding and signaling molecules have been examined using melon seedlings at the four-leaf stage (S5 and S6 Figs). Real-time quantitative PCR analysis showed that the expression level of *CmLOX10* were extensively up-regulated by mechanical wounding and exogenous application of MeJA and H_2_O_2_. The phenomenon that *CmLOX10* expression is induced by MeJA but suppressed by SA has been suggested that JA and SA have antagonistic effects in defense responses [48–50]. In contrast, the expression level of *CmLOX13* was extensively up-regulated by mechanical wounding and exogenous application of H_2_O_2_ as same as *CmLOX10*, but the gene was induced by SA and not responsive to MeJA. Based on these results, we suggest that CmLOX10 and CmLOX13 may involve in different plant signals in vivo. These data above will be helpful for us in understanding their roles in oriental melon development and defense responses. However, the further study is needed to better understand the roles of CmLOX10 and CmLOX13 in oriental melon.

## Conclusions

We cloned the two full-length clones of *CmLOX10* and *CmLOX13* from oriental melon which they have a high degree of identity at the amino acids. Then they were expressed as recombinant proteins and assayed in vitro. Recombinant His-tagged CmLOX10 and CmLOX13 showed 13-LOX activity and preferentially used linoleic acid as substrate. Subcellular localization analysis by transient expression in tobacco leaves showed that CmLOX10 was located in plasma membrane, while CmLOX13 was located in chloroplasts. Combining the results with the phylogenetic tree analysis of the two LOXs, it could be predicted that CmLOX10 and CmLOX13 encode a 13-LOX probably involved in the production of C6 volatile compounds and have different locations which may play different functions during plant growth and development. In conclusion, such studies will help us to better understand the functions of LOXs both in development and the defense response in oriental melon.

## Supporting Information

S1 Fig Alignment of the nucleotide sequences of *CmLOX10* isolated from oriental melon (*Cucumis melo* var. *makuwa* Makino) and *GeLOX10* from the melon (*Cucumis melon* L.) genome database. A TAA stop codon upstream from the first ATG of the ORF of *CmLOX10* is framed. The locations of the three gene-specific primers for 5’ RACE of the *CmLOX10* which are GSP1, GSP2 and GSP3 are single, double and triple underlined, respectively. The seven different nucleotides between the *CmLOX10* and *GeLOX10* are indicated by asterisks.
(DOCX)

S2 Fig Alignment of the nucleotide sequences of *CmLOX13* isolated from oriental melon (*Cucumis melo* var. *makuwa* Makino) and *GeLOX13* from the melon (*Cucumis melon* L.) genome database. The ATG of the ORF of *CmLOX13* is framed. The locations of the three gene-specific primers for 5’ RACE of the *CmLOX10* which are GSP1, GSP2 and GSP3 are single, double and triple underlined, respectively. The five different nucleotides between the *CmLOX13* and *GeLOX13* are indicated by asterisks.
(DOCX)

S3 Fig Schematic representation of the conserved domains of CmLOX10 and CmLOX13 by the NCBI Conserved Domain Search program. (TIF)

S4 Fig The expression analysis of *CmLOX10* and *CmLOX13* in different tissues of oriental melon by Semi-quantitative and Real-time RT-PCR. The samples identities are as follows: 1, root; 2, stem; 3, young leaf; 4, female flower; 5, male flower; 6, seeds without soaking; 7, seeds were soaked in tap water for 24 h; 8, seeds were soaked in tap water for 72 h; 9, 5d after pollination (DAP); 10, 10 DAP; 11, 15 DAP; 12, 20 DAP; 13, 25 DAP; 14, 30 DAP; 15, 35 DAP; 16, 40 DAP.
(TIF)

S5 Fig Relative expression analysis of *CmLOX10* in response to mechanical wounding and signaling molecules was determined by real-time RT-PCR. (TIF)

S6 Fig Relative expression analysis of CmLOX13 in response to mechanical wounding and signaling molecules was determined by real-time RT-PCR (TIF)

S1 Table Primer sequences used in the study. Nucleotides underlined were used for producing *Eco*RI and *Xho*I restriction site.
(DOCX)

## References

[1] Feussner I, Wasternack C. The lipoxygenase pathway. Annual review of plant biology. 2002; 53: 275–97. 10.1146/annurev.arplant.53.100301.135248 12221977

[2] Liavonchanka A, Feussner I. Lipoxygenases: occurrence, functions and catalysis. Journal of plant physiology. 2006; 163(3): 348–57. 10.1016/j.jplph.2005.11.006 16386332

[3] Zhu Z, Qian F, Yang R, Chen J, Luo Q, Chen H, et al A lipoxygenase from red alga *Pyropia haitanensis*, a unique enzyme catalyzing the free radical reactions of polyunsaturated fatty acids with triple ethylenic bonds. PLOS One. 2015; 10(2): e0117351 10.1371/journal.pone.0117351 25658744 PMC4319731

[4] Lohelaid H, Teder T, Toldsepp K, Ekins M, Samel N. Up-regulated expression of AOS-LOXa and increased eicosanoid synthesis in response to coral wounding. PLOS One. 2014; 9(2): e89215 10.1371/journal.pone.0089215 24551239 PMC3925239

[5] Brodhun F, Cristobal-Sarramian A, Zabel S, Newie J, Hamberg M, Feussner I. An iron 13S-lipoxygenase with an alpha-linolenic acid specific hydroperoxidase activity from *Fusarium oxysporum*. PLOS One. 2013; 8(5): e64919 10.1371/journal.pone.0064919 23741422 PMC3669278

[6] Andreou A, Feussner I. Lipoxygenases—Structure and reaction mechanism. Phytochemistry. 2009; 70(13–14): 1504–10. 10.1016/j.phytochem.2009.05.008 19767040

[7] Porta H, Rocha-Sosa M. Plant lipoxygenases. Physiological and molecular features. Plant physiology. 2002; 130(1): 15–21. 10.1104/pp.010787 12226483 PMC1540254

[8] Howe G, Schilmiller A. Oxylipin metabolism in response to stress. Current Opinion in Plant Biology. 2002; 5(3): 230–6. 11960741 10.1016/s1369-5266(02)00250-9

[9] Blée E. Impact of phyto-oxylipins in plant defense. Trends in Plant Science. 2002; 7(7): 315–22. 12119169 10.1016/s1360-1385(02)02290-2

[10] Wasternack C, Hause B. Jasmonates and octadecanoids: signals in plant stress responses and development. Progress in Nucleic Acid Research and Molecular Biology. 2002; 72: 165–221. 12206452 10.1016/s0079-6603(02)72070-9

[11] Liu S, Han B. Differential expression pattern of an acidic 9/13-lipoxygenase in flower opening and senescence and in leaf response to phloem feeders in the tea plant. BMC plant biology. 2010; 10: 228 10.1186/1471-2229-10-228 20969806 PMC3095316

[12] Palmieri-Thiers C, Canaan S, Brunini V, Lorenzi V, Tomi F, Desseyn JL, et al A lipoxygenase with dual positional specificity is expressed in olives (Olea europaea L.) during ripening. Biochimica et biophysica acta. 2009; 1791(5): 339–46. 10.1016/j.bbalip.2009.02.012 19268561

[13] Wang R, Shen W, Liu L, Jiang L, Liu Y, Su N, et al A novel lipoxygenase gene from developing rice seeds confers dual position specificity and responds to wounding and insect attack. Plant molecular biology. 2008; 66(4): 401–14. 10.1007/s11103-007-9278-0 18185911

[14] Hwang IS, Hwang BK. The pepper 9-lipoxygenase gene *CaLOX1* functions in defense and cell death responses to microbial pathogens. Plant physiology. 2010; 152(2): 948–67. 10.1104/pp.109.147827 19939946 PMC2815858

[15] Mariutto M, Duby F, Adam A, Bureau C, Fauconnier ML, Ongena M, et al The elicitation of a systemic resistance by *Pseudomonas putida* BTP1 in tomato involves the stimulation of two lipoxygenase isoforms. BMC plant biology. 2011; 11: 29 10.1186/1471-2229-11-29 21294872 PMC3042376

[16] Veronico P, Giannino D, Melillo MT, Leone A, Reyes A, Kennedy MW, et al A novel lipoxygenase in pea roots. Its function in wounding and biotic stress. Plant physiology. 2006; 141(3): 1045–55. 10.1104/pp.106.081679 16679421 PMC1489892

[17] Kolomiets M, Hannapel D, Chen H, Tymeson M, Gladon R. Lipoxygenase is involved in the control of potato tuber development. Plant Cell. 2001; 13(3): 613–26. 11251100 10.1105/tpc.13.3.613 PMC135504

[18] Chen G, Hackett R, Walker D, Taylor A, Lin Z, Grierson D. Identification of a specific isoform of tomato lipoxygenase (TomloxC) involved in the generation of fatty acid-derived flavor compounds. Plant physiology. 2004; 136(1): 2641–51. 10.1104/pp.104.041608 15347800 PMC523329

[19] Acosta IF, Laparra H, Romero SP, Schmelz E, Hamberg M, Mottinger JP, et al tasselseed1 is a lipoxygenase affecting jasmonic acid signaling in sex determination of maize. Science. 2009; 323(5911): 262–5. 10.1126/science.1164645 19131630

[20] Seltmann MA, Stingl NE, Lautenschlaeger JK, Krischke M, Mueller MJ, Berger S. Differential Impact of Lipoxygenase 2 and Jasmonates on Natural and Stress-Induced Senescence in Arabidopsis. Plant physiology. 2010; 152(4): 1940–50. 10.1104/pp.110.153114 20190093 PMC2850018

[21] Porta H, Figueroa-Balderas RE, Rocha-Sosa M. Wounding and pathogen infection induce a chloroplast-targeted lipoxygenase in the common bean (Phaseolus vulgaris L.). Planta. 2008; 227(2): 363–73. 10.1007/s00425-007-0623-y 17899174

[22] Padilla MN, Hernandez ML, Sanz C, Martinez-Rivas JM. Stress-dependent regulation of 13-lipoxygenases and 13-hydroperoxide lyase in olive fruit mesocarp. Phytochemistry. 2014; 102: 80–8. 10.1016/j.phytochem.2014.01.024 24629805

[23] Gao X, Stumpe M, Feussner I, Kolomiets M. A novel plastidial lipoxygenase of maize (Zea mays) ZmLOX6 encodes for a fatty acid hydroperoxide lyase and is uniquely regulated by phytohormones and pathogen infection. Planta. 2008; 227(2): 491–503. 10.1007/s00425-007-0634-8 17922288

[24] Li S-t, Zhang M, Fu C-h, Xie S, Zhang Y, Yu L-j. Molecular Cloning and Characterization of Two 9-Lipoxygenase Genes from *Taxus chinensis*. Plant Molecular Biology Reporter. 2012; 30(6): 1283–90. 10.1007/s11105-012-0439-1

[25] Chen Z, Chen X, Yan H, Li W, Li Y, Cai R, et al The Lipoxygenase Gene Family in Poplar: Identification, Classification, and Expression in Response to MeJA Treatment. PLOS One. 2015; 10(4): e0125526 10.1371/journal.pone.0125526 25928711 PMC4415952

[26] Garcia-Mas J, Benjak A, Sanseverino W, Bourgeois M, Mir G, González V, et al The genome of melon (*Cucumis melo* L.). Proceedings of the National Academy of Sciences of the United States of America. 2012; 109(29): 11872–7. 10.1073/pnas.1205415109 22753475 PMC3406823

[27] Zhang C, Jin Y, Liu J, Tang Y, Cao S, Qi H. The phylogeny and expression profiles of the lipoxygenase (LOX) family genes in the melon (*Cucumis melo* L.) genome. Scientia Horticulturae. 2014; 170: 94–102. 10.1016/j.scienta.2014.03.005

[28] Simpson R. Purifying Proteins for Proteomics: A Laboratory Manual. United States: Cold Spring Harbor Laboratory Press; 2003.

[29] Gata J, Pinto M, Macías P. Lipoxygenase activity in pig muscle: purification and partial characterization. Journal of agricultural and food chemistry. 1996; 44(9): 2573–7.

[30] Long Q, Zhang W, Wang P, Shen W, Zhou T, Liu N, et al Molecular genetic characterization of rice seed lipoxygenase 3 and assessment of its effects on seed longevity. Journal of Plant Biology. 2013; 56(4): 232–42. 10.1007/s12374-013-0085-7

[31] Karimi M, Inzé D, Depicker A. GATEWAY vectors for *Agrobacterium*-mediated plant transformation. Trends in Plant Science. 2002; 7(5): 193–5. 11992820 10.1016/s1360-1385(02)02251-3

[32] Padilla MN, Hernandez ML, Sanz C, Martinez-Rivas JM. Molecular cloning, functional characterization and transcriptional regulation of a 9-lipoxygenase gene from olive. Phytochemistry. 2012; 74: 58–68. 10.1016/j.phytochem.2011.11.006 22169502

[33] Coffa G, Brash AR. A single active site residue directs oxygenation stereospecificity in lipoxygenases: stereocontrol is linked to the position of oxygenation. Proceedings of the National Academy of Sciences of the United States of America. 2004; 101(44): 15579–84. 10.1073/pnas.0406727101 15496467 PMC524819

[34] Caldelari D, Wang G, Farmer EE, Dong X. Arabidopsis *lox3 lox4* double mutants are male sterile and defective in global proliferative arrest. Plant molecular biology. 2011; 75(1–2): 25–33. 10.1007/s11103-010-9701-9 21052784

[35] Farmaki T, Sanmartin M, Jimenez P, Paneque M, Sanz C, Vancanneyt G, et al Differential distribution of the lipoxygenase pathway enzymes within potato chloroplasts. Journal of experimental botany. 2007; 58(3): 555–68. 10.1093/jxb/erl230 17210991

[36] Padilla MN, Hernandez ML, Sanz C, Martinez-Rivas JM. Functional characterization of two 13-lipoxygenase genes from olive fruit in relation to the biosynthesis of volatile compounds of virgin olive oil. Journal of agricultural and food chemistry. 2009; 57(19): 9097–107. 10.1021/jf901777j 19722522

[37] Podolyan A, White J, Jordan B, Winefield C. Identification of the lipoxygenase gene family from *Vitis vinifera* and biochemical characterisation of two 13-lipoxygenases expressed in grape berries of Sauvignon Blanc. Functional Plant Biology. 2010; 37: 767–84.

[38] Hornung E, Walther M, Feussner I. Conversion of cucumber linoleate 13-lipoxygenase to a 9-lipoxygenating species by site-directed mutagenesis. Proceedings of the National Academy of Sciences of the United States of America. 1999; 96(7): 4192–7. 10097186 10.1073/pnas.96.7.4192 PMC22443

[39] Coffa G, Schneider C, Brash AR. A comprehensive model of positional and stereo control in lipoxygenases. Biochemical and biophysical research communications. 2005; 338(1): 87–92. 10.1016/j.bbrc.2005.07.185 16111652

[40] Siedow J. Plant lipoxygenases: structure and functions. Annual review of plant physiology and plant molecular biology. 1991; 42: 145–88.

[41] Schwab W, Davidovich-Rikanati R, Lewinsohn E. Biosynthesis of plant-derived flavor compounds. The Plant journal. 2008; 54(4): 712–32. 10.1111/j.1365-313X.2008.03446.x 18476874

[42] Leon J, Royo J, Vancanneyt G, Sanz C, Silkowski H, Griffiths G, et al Lipoxygenase H1 gene silencing reveals a specific role in supplying fatty acid hydroperoxides for aliphatic aldehyde production. The Journal of biological chemistry. 2002; 277(1): 416–23. 10.1074/jbc.M107763200 11675388

[43] Zhou G, Qi J, Ren N, Cheng J, Erb M, Mao B, et al Silencing *OsHI-LOX* makes rice more susceptible to chewing herbivores, but enhances resistance to a phloem feeder. The Plant journal. 2009; 60(4): 638–48. 10.1111/j.1365-313X.2009.03988.x 19656341

[44] Halitschke R, Baldwin I. Antisense LOX expression increases herbivore performance by decreasing defense responses and inhibiting growth-related transcriptional reorganization in *Nicotiana attenuata*. The Plant journal. 2003; 36(6): 794–807. 14675445 10.1046/j.1365-313x.2003.01921.x

[45] Christensen SA, Nemchenko A, Borrego E, Murray I, Sobhy IS, Bosak L, et al The maize lipoxygenase, *ZmLOX10*, mediates green leaf volatile, jasmonate and herbivore-induced plant volatile production for defense against insect attack. The Plant journal. 2013; 74(1): 59–73. 10.1111/tpj.12101 23279660

[46] Bate N, Rothstein S. C6-volatiles derived from the lipoxygenase pathway induce a subset of defense-related genes. The Plant journal. 1998; 16(5): 561–9. 10036774 10.1046/j.1365-313x.1998.00324.x

[47] Prost I, Dhondt S, Rothe G, Vicente J, Rodriguez MJ, Kift N, et al Evaluation of the antimicrobial activities of plant oxylipins supports their involvement in defense against pathogens. Plant physiology. 2005; 139(4): 1902–13. 10.1104/pp.105.066274 16299186 PMC1310568

[48] Peña-Cortes H, Albrecht T, Prat S, Weiler E, Willmitzer L. Aspirin prevents wound-induced gene expression in tomato leaves by blocking jasmonic acid biosynthesis. Planta. 1993; 191: 123–8.

[49] Feys B, Parker J. Interplay of signaling pathways in plant disease resistance. Trends in Genetics. 2000; 16(10): 449–55. 11050331 10.1016/s0168-9525(00)02107-7

[50] Cipollini D, Enright S, Traw MB, Bergelson J. Salicylic acid inhibits jasmonic acid-induced resistance of *Arabidopsis thaliana* to *Spodoptera exigua*. Molecular ecology. 2004; 13(6): 1643–53. 10.1111/j.1365-294X.2004.02161.x 15140107

