# Supplementary figures and images for: Heterologous Expression and Biochemical Characterization of Two Lipoxygenases in Oriental Melon, Cucumis melo var. makuwa Makino
Source: PLoS One. 2016 Apr 21;11(4):e0153801. doi: 10.1371/journal.pone.0153801 (PMC4839669; doi:10.1371/journal.pone.0153801)

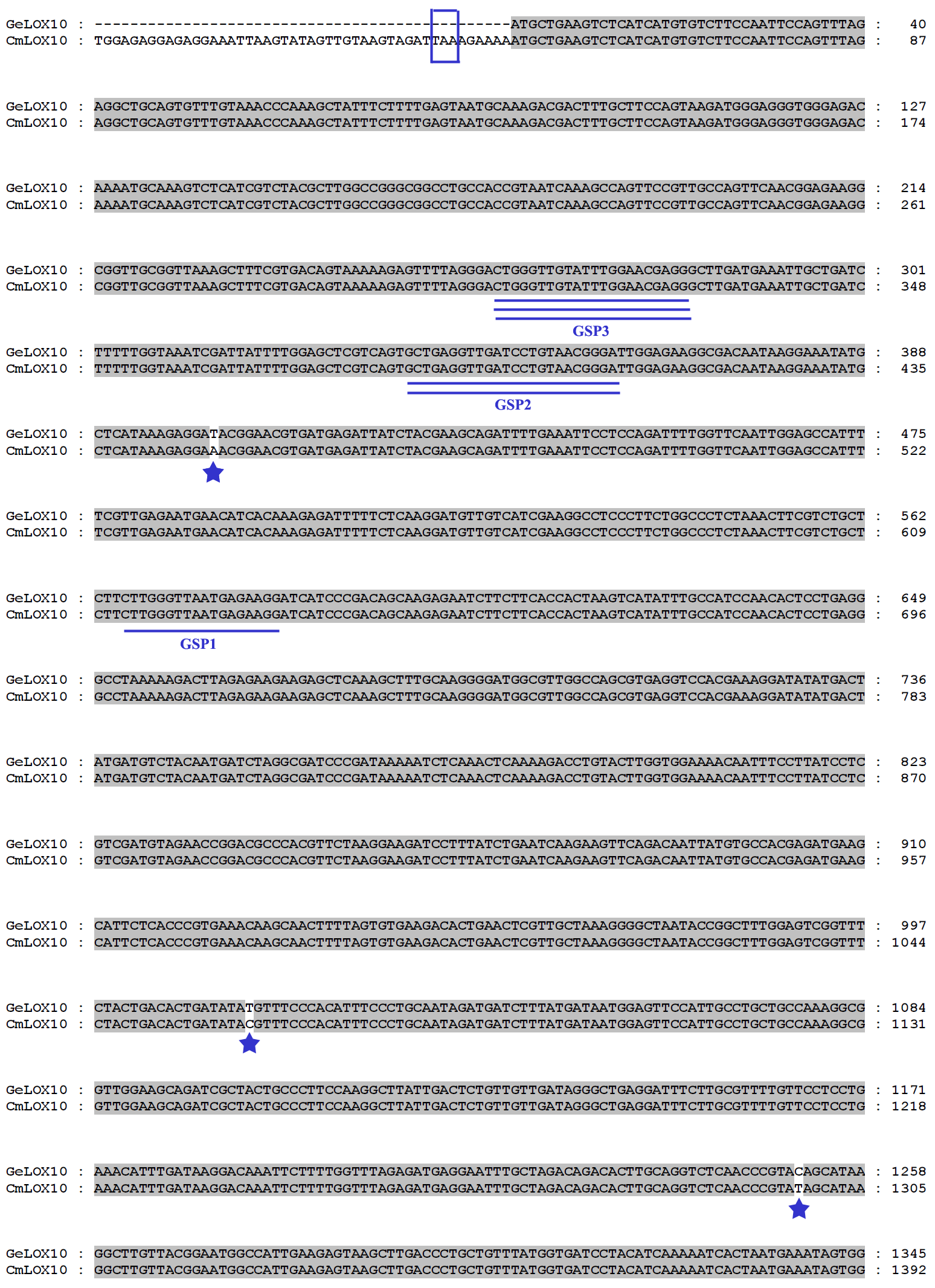


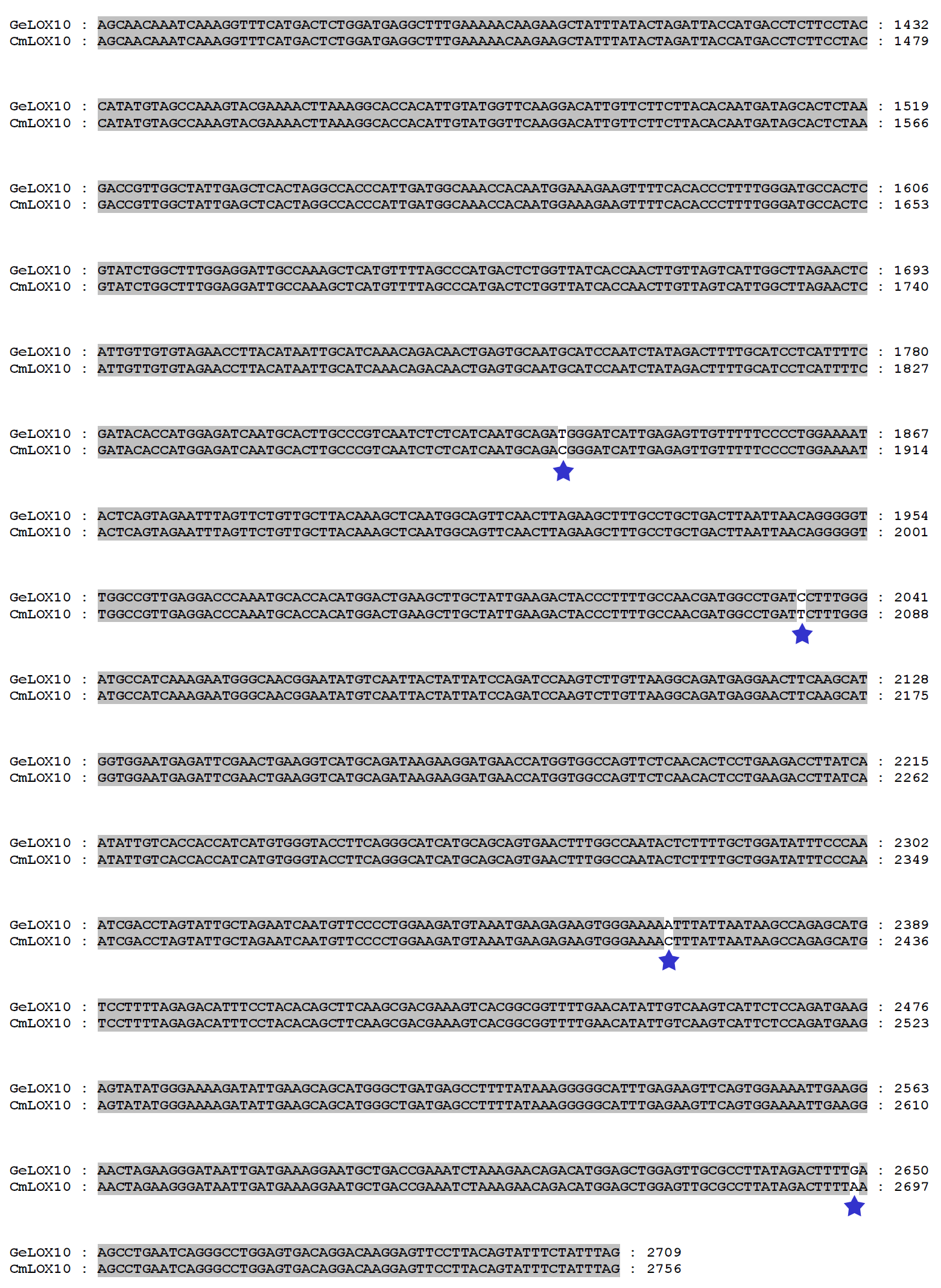

Supplement: S1 Fig — A TAA stop codon upstream from the first ATG of the ORF of CmLOX10 is framed. The locations of the three gene-specific primers for 5’ RACE of the CmLOX10 which are GSP1, GSP2 and GSP3 are single, double and triple underlined, respectively. The seven different nucleotides between the CmLOX10 and GeLOX10 are indicated by asterisks. (DOCX) [file pone.0153801.s001.docx]

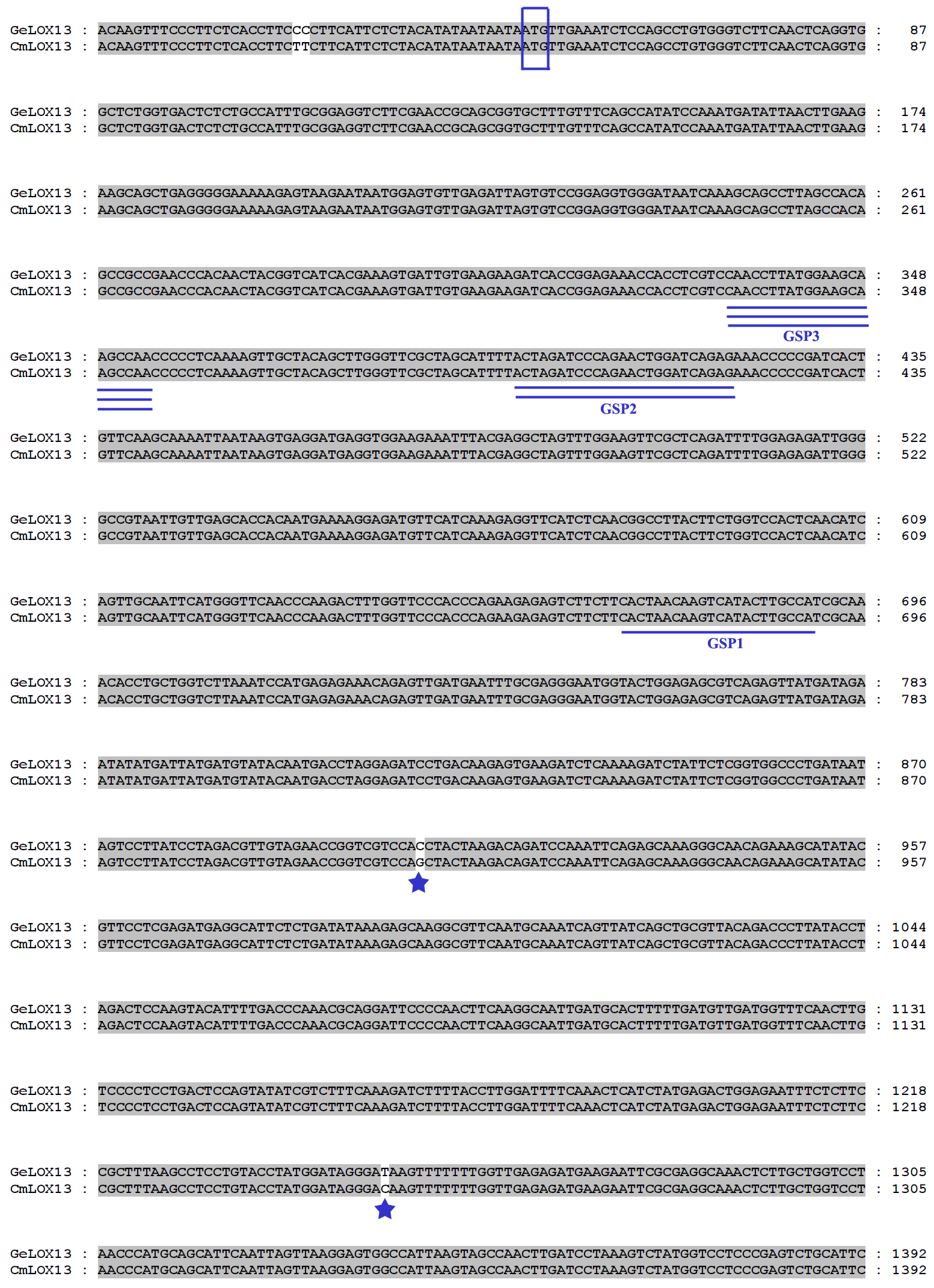


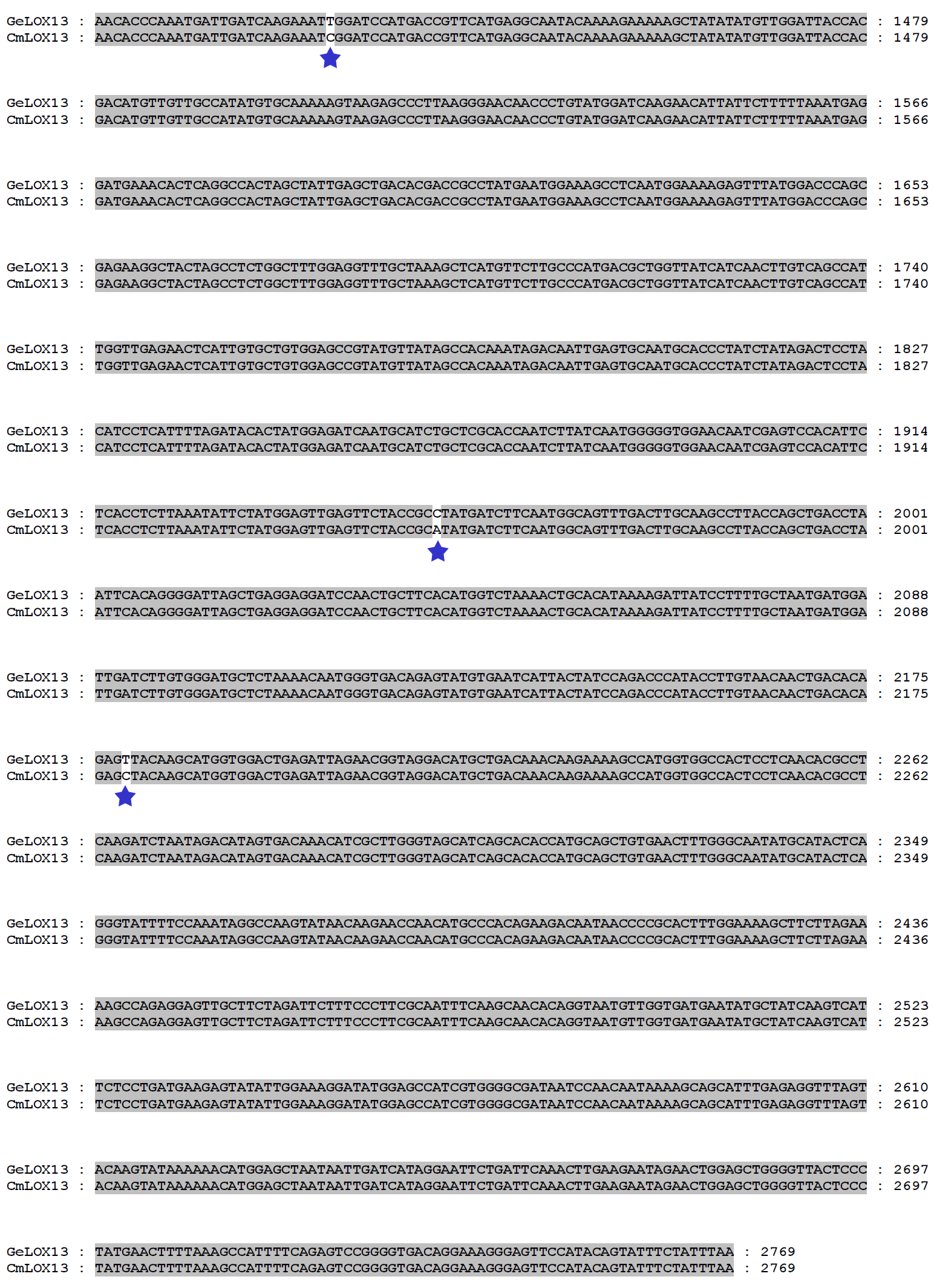

Supplement: S2 Fig — The ATG of the ORF of CmLOX13 is framed. The locations of the three gene-specific primers for 5’ RACE of the CmLOX10 which are GSP1, GSP2 and GSP3 are single, double and triple underlined, respectively. The five different nucleotides between the CmLOX13 and GeLOX13 are indicated by asterisks. (DOCX) [file pone.0153801.s002.docx]

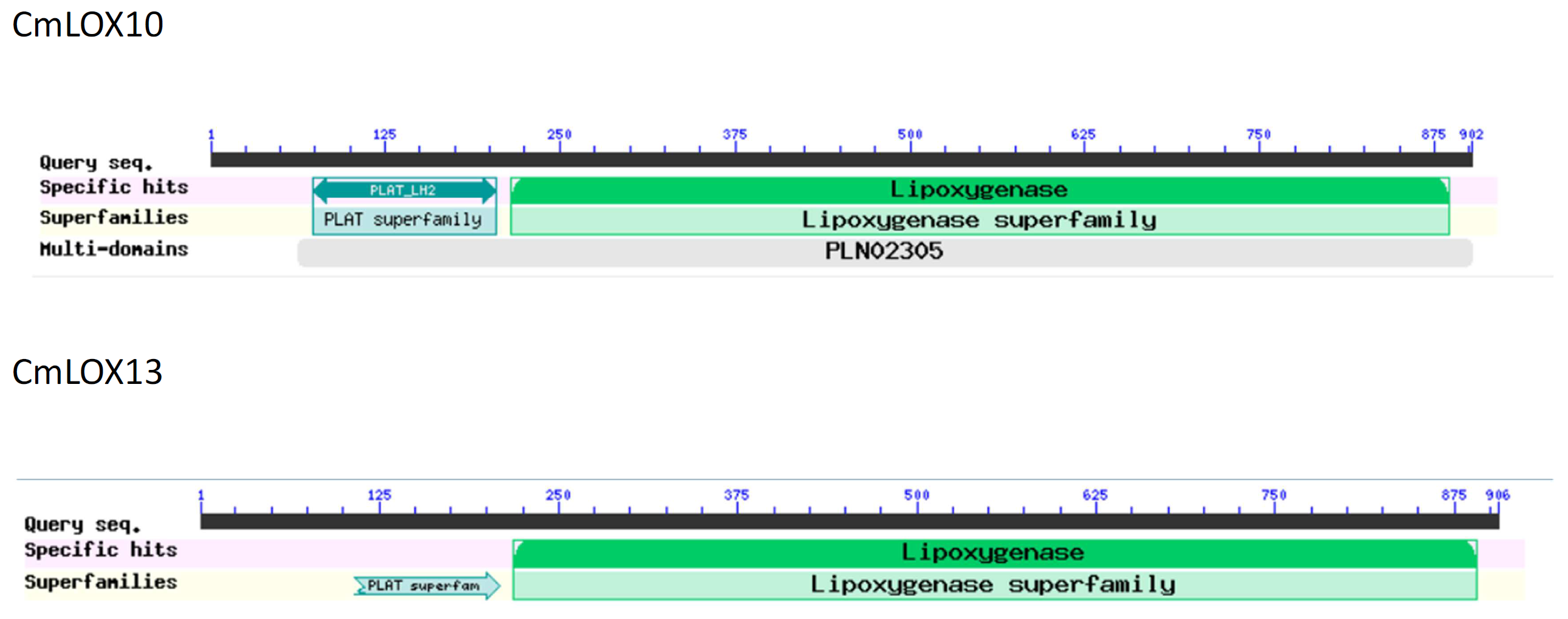

Supplement: S3 Fig — (TIF) [file pone.0153801.s003.tif]

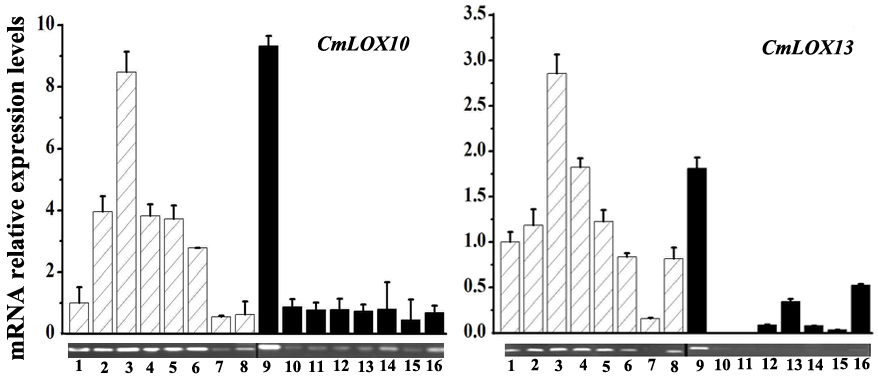

Supplement: S4 Fig — The samples identities are as follows: 1, root; 2, stem; 3, young leaf; 4, female flower; 5, male flower; 6, seeds without soaking; 7, seeds were soaked in tap water for 24 h; 8, seeds were soaked in tap water for 72 h; 9, 5d after pollination (DAP); 10, 10 DAP; 11, 15 DAP; 12, 20 DAP; 13, 25 DAP; 14, 30 DAP; 15, 35 DAP; 16, 40 DAP. (TIF) [file pone.0153801.s004.tif]

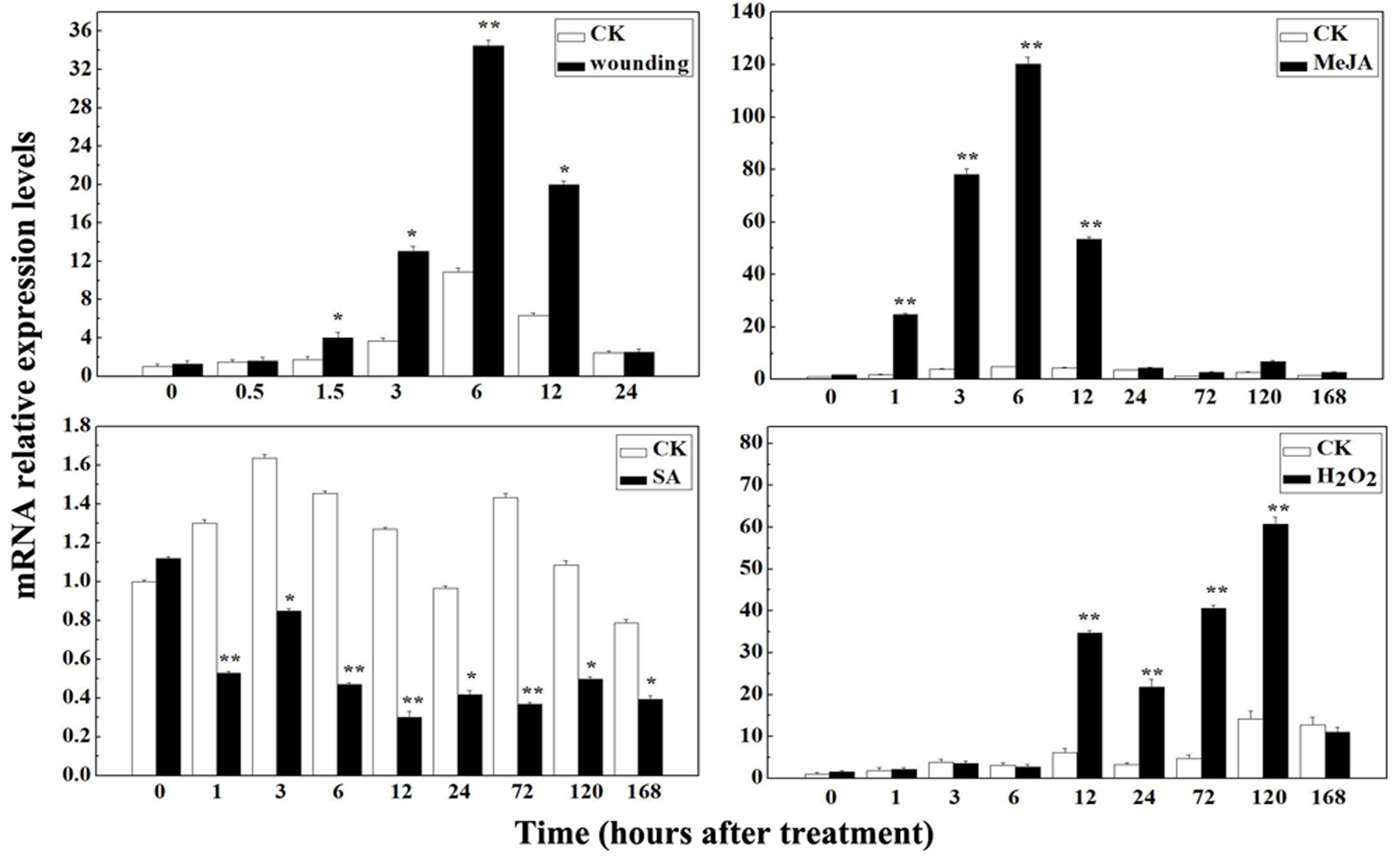

Supplement: S5 Fig — (TIF) [file pone.0153801.s005.tif]

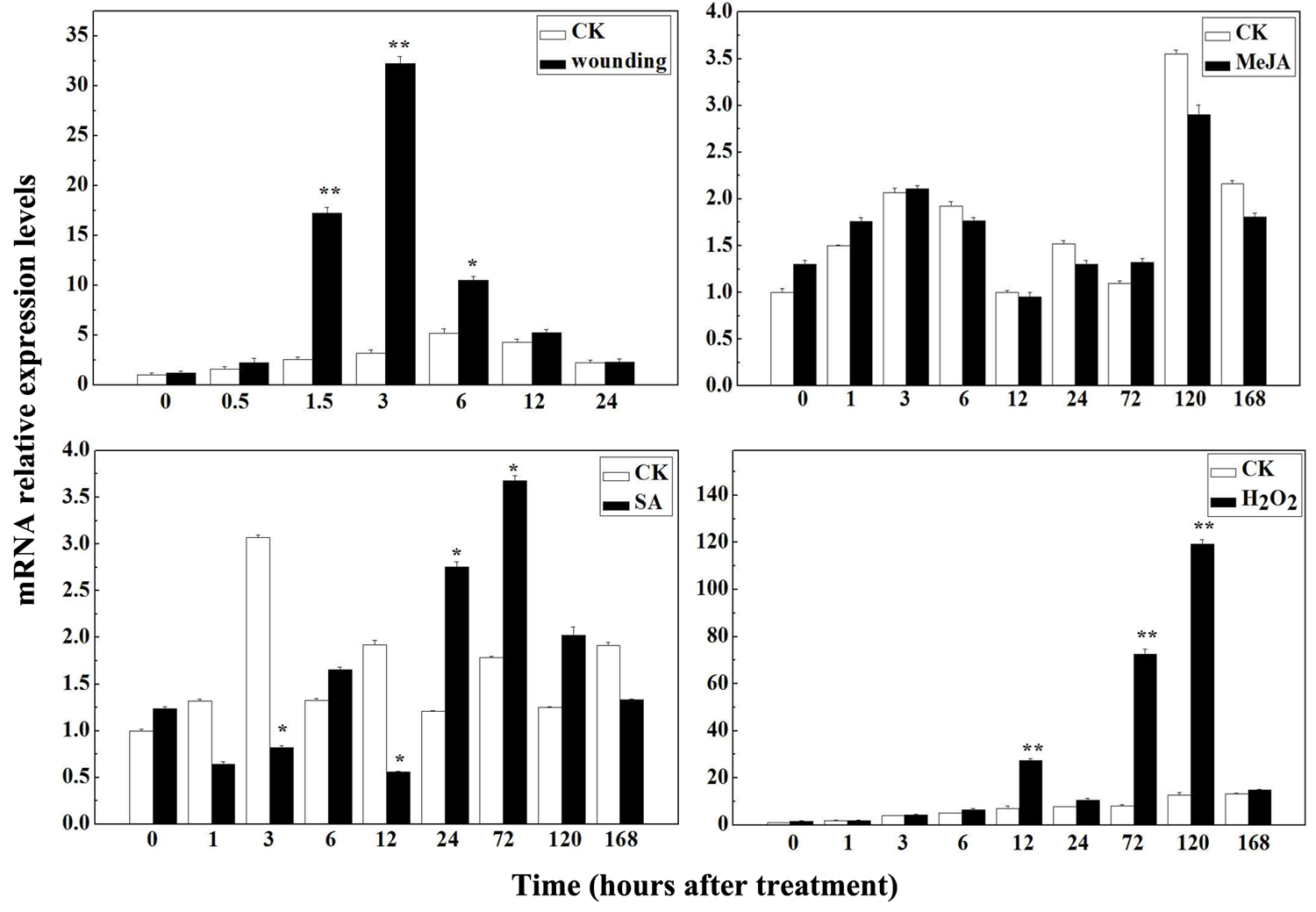

Supplement: S6 Fig — (TIF) [file pone.0153801.s006.tif]
