# Supplementary material for: Heterologous Expression and Biochemical Characterization of Two Lipoxygenases in Oriental Melon, Cucumis melo var. makuwa Makino
Source: PLoS One. 2016 Apr 21;11(4):e0153801. doi: 10.1371/journal.pone.0153801 (PMC4839669; doi:10.1371/journal.pone.0153801)
Supplement: S1 Table — Nucleotides underlined were used for producing EcoRI and XhoI restriction site. (DOCX) [file pone.0153801.s007.docx]

**Table. S1 Primer sequences used in the study.** Nucleotides underlined were used for producing *Eco*RI and *Xho*I restriction site.

| Primers | Sequence (5’-3’) |
| --- | --- |
| CmLOX10-F | ATGCTGAAGTCTCATCATGTG |
| CmLOX10-R | TGTTTCTGAGTGGTTTGG |
| CmLOX13-F | TCTCACCTTCCCCTTCATT |
| CmLOX13-R | TCATCTGGCCTTTGTCATT |
| LOX10-GSP1 | CCTTCTCATTAACCCAAG |
| LOX10-GSP2 | TCCCGTTACAGGATCAACCTCAGC |
| LOX10-GSP3 | CCTCGTTCCAAATACAACCCAG |
| LOX13-GSP1 | TGGCAAGTATGACTTGTTAGTG |
| LOX13-GSP2 | CTCTGATCCAGTTCTGGGATCTAGT |
| LOX13-GSP3 | TTGGCTTGCTTCCATAAGGTTG |
| LOX13t-F-1 | AATTCTCTTCGAACCGCAGCGGT |
| LOX13t-R-1 | GTTAAATAGAAATACTGTATGGAACTCCC |
| LOX13t-F-2 | CTCTTCGAACCGCAGCGGT |
| LOX13t-R-2 | TCGAGTTAAATAGAAATACTGTATGGAACTCCC |
| LOX10t-F-1 | AATTCTGGGAGGGTGGGAGACAAAAT |
| LOX10t-R-1 | GCTAAATAGAAATACTGTAAGGAACTC |
| LOX10t-F-2 | CTGGGAGGGTGGGAGACAAAAT |
| LOX10t-R-2 | TCGAGCTAAATAGAAATACTGTAAGGAACTC |
| LOX10f-F-1 | AATTCATGCTGAAGTCTCATCATGTGT |
| LOX10f-R-1 | GCTAAATAGAAATACTGTAAGGAACTC |
| LOX10f-F-2 | CATGCTGAAGTCTCATCATGTGT |
| LOX10f-R-2 | TCGAGCTAAATAGAAATACTGTAAGGAACTC |
| LOX10G-F  LOX10G-R  LOX13G-F  LOX13G-R | CACCATGCTGAAGTCTCATCATGTGT    AATAGAAATACTGTAAGGAACTCCT  CACCATGTTGAAATCTCCAGCCTGT  AATAGAAATACTGTATGGAACTCCC' |
